# Serological insights from SARS-CoV-2 heterologous prime and boost responses in Thailand

**DOI:** 10.1038/s41598-024-84392-2

**Published:** 2025-01-09

**Authors:** Daniel Ward, Lapasrada Pattarapreeyakul, Rujiraporn Pitaksalee, Naphatcha Thawong, Waritta Sawaengdee, Suthida Tuntigumthon, Catriona Patterson, Kevin Tetteh, Susana Campino, Panadda Dhepakson, Surakameth Mahasirimongkol, Taane G. Clark

**Affiliations:** 1 Faculty of Infectious and Tropical Diseases, London School of Hygiene and Tropical Medicine (LSHTM), Keppel Street, London, WC1E 7HT UK; 2 Department of Medical Sciences, Medical Life Sciences Institute, Ministry of Public Health, 88/7 Tiwanon Road, Nonthaburi, 11000 Thailand; 3 Faculty of Epidemiology and Population Health, LSHTM, London, UK

**Keywords:** SARS-CoV-2, COVID-19, Vaccine, Antibody

## Abstract

During the COVID-19 pandemic, heterologous vaccination strategies were employed to alleviate the strain on vaccine supplies. The Thailand Ministry of Health adopted these strategies using vector, inactivated, and mRNA vaccines. However, this approach has introduced challenges for SARS-CoV-2 sero-epidemiology studies. Our study analysed 647 samples from healthcare workers who received CoronaVac, ChAdOx1 nCoV-19, and BNT162b2 vaccines. The serological profile encompassed responses to various SARS-CoV-2 variants and vectors, measuring IgG, IgM, and IgA isotypes, alongside IgG avidity assays. The results demonstrated that heterologous CoronaVac/ChAdOx1 nCoV-19 schedules elicited significantly stronger antibody responses compared to homologous schedules (IgG: 1.2-fold, IgM: 10.9-fold, IgA: 3.1-fold increase). Additionally, a heterologous BNT162b2 boost at 4-weeks post-initial vaccination showed greater antibody levels than a ChAdOx1 nCoV-19 boost (IgG: 1.1-fold, IgM: slight decrease, IgA: 1.5-fold increase). Using a combination of three analytes, IgG against wild-type Spike trimer, nucleoprotein and Omicron receptor binding domains, enabled the clustering of responses within a statistical Gaussian mixture model that successfully discriminates between breakthrough infections and vaccination types (F-score = 0.82). The development of statistical models to predict breakthrough infections can improve serological surveillance. Overall, our study underscores the necessity for vaccine (re-)development and the creation of serological tools to monitor vaccine performance.

**Supplementary Information:**

The online version contains supplementary material available at 10.1038/s41598-024-84392-2.

## Introduction

The coronavirus disease 2019 (COVID-19) pandemic, caused by SARS-CoV-2, prompted an unprecedented surge in biomedical research yielding seven vaccines that were approved by the Thailand Food and Drug Thailand Administration (FDA), amongst other global counterparts, before the end of year 2022. Throughout 2020, numerous vaccine candidate frontrunners emerged employing a range of technologies, namely using mRNA (mRNA-1273 and BNT162b2)^1,2^, adenovirus vector (ChAdOx1 nCoV-19, Sputnik)^3,4^, and whole inactivated virus (CoronaVac) platforms^5^. To relieve strain on overstretched vaccine supply chains, governments worldwide authorised the trial of heterologous primary and boost vaccine programmes, a practice which was validated by preliminary studies^6^, and later confirmed by large, well-controlled clinical trials^7^. In turn, to reduce the surge in SARS-CoV-2 morbidity, particularly amongst healthcare workers in Bangkok, in July 2021, Thailand FDA approved the use of heterologous primary and boost CoronaVac (Sinovac Biotech) / ChAdOx1 nCoV-19 (AstraZeneca, University of Oxford) vaccination and later CoronaVac / BNT162b2 (BioNTech, Pfizer) programmes (Figure S1).

Prior to the COVID-19 pandemic, the use of a heterologous regimen to enhance immunogenicity was trialled as an Ebola virus control strategy to enhance the longevity of immunity in regions where a reactive ring-fence strategy was not feasible^8,9^. Current research regarding heterologous primary/boost SARS-CoV-2 vaccine regimen emphasises its safety^7,8,10^. Heterologous vaccination schedules involving vectored (ChAdOx1 nCoV-19/Ad26.COV2.S), inactivated (CoronaVac/Covaxin) and mRNA vaccine (BNT162b2/mRNA-1273) platforms often yielded enhanced antibody responses when compared with homologous inactivated or vectored schedules^10,11^. Moreover, cellular responses were found to be enhanced in heterologous recipients compared with that of homologous vectored and mRNA vaccines^12,13^. Fewer studies have covered heterologous primary regimens with inactivated vaccines but, those which have reported findings, suggest diminished responses compared with homologous vectored or mRNA platforms^10^. Moreover, the protection elicited by wild-type SARS-CoV-2 vaccines is reduced in the context of Omicron variant infections^14,15^. Therefore, the analysis of mixed vaccine regimens may yield insights into the control of future variants.

Studies on vaccine uptake and population immunity throughout the COVID-19 pandemic played a central role in control efforts^16^. Seroprevalence surveys were used to understand the prevalence of disease by detecting host antibody responses. The same analyses were used to understand the prevalence of immunisation within a community, guiding future control efforts for vulnerable populations. The differentiation of vaccine-specific responses as opposed to natural SARS-CoV-2 or breakthrough infections was straightforward, largely due to the presence of responses specific to other (non-spike) structural and non-structural components of the SARS-CoV-2 virus, for example, the nucleoprotein, enabling a differential analysis^17^. However, those immunised with an inactivated virus platform will harbour all viral immunogens, confounding assays that would otherwise have differentiated vaccination from infection. The ability to model host antibody responses in the setting of heterologous vaccination and breakthrough infection yields greater insights into the efficacy and longevity and of vaccination responses in each population, facilitating the allocation of resources where they are most needed. This strategy has been applied previously for monitoring measles, mumps and rubella immunity^18^ and human papillomavirus^19^, with other studies offering insights into heterogeneity of responses to vaccination within a population^20^.

Blood-based measurements (e.g., cell counts, antibody or antigen detection) can be utilised to classify immunological status into categories such as positive, intermediate, and negative^21,22^. A popular method for this classification is Gaussian Mixture Models (GMMs), a probabilistic approach that clusters data by modelling it as a mixture of multiple normally distributed components, each representing a distinct subgroup. Recent applications of GMMs include classifying long COVID-19 severity based on persistent symptoms^23^ and predicting new COVID-19 case trends across various countries and time periods^24^. In this study, GMMs will be used to cluster individuals by their antibody profiles in response to different vaccination regimens, enabling us to identify distinct immunological patterns and better understand the diversity of immune responses elicited by these regimens.

Here, we report the findings of an investigation of antibody profiles after prime and boost with homologous and heterologous vaccination regimens in a Thai population. Through using a customised multiplex microsphere assay for spike, nucleoprotein and viral vector antigens, IgG, IgM, IgA and IgG-avidity responses, the antibody dynamics across a cohort of 415 healthcare workers are described. Using the complex dataset, a GMM was developed to classify the vaccine and infection status of participants.

## Results

### Description of vaccine recipients in the study population

**Fig. 1:**
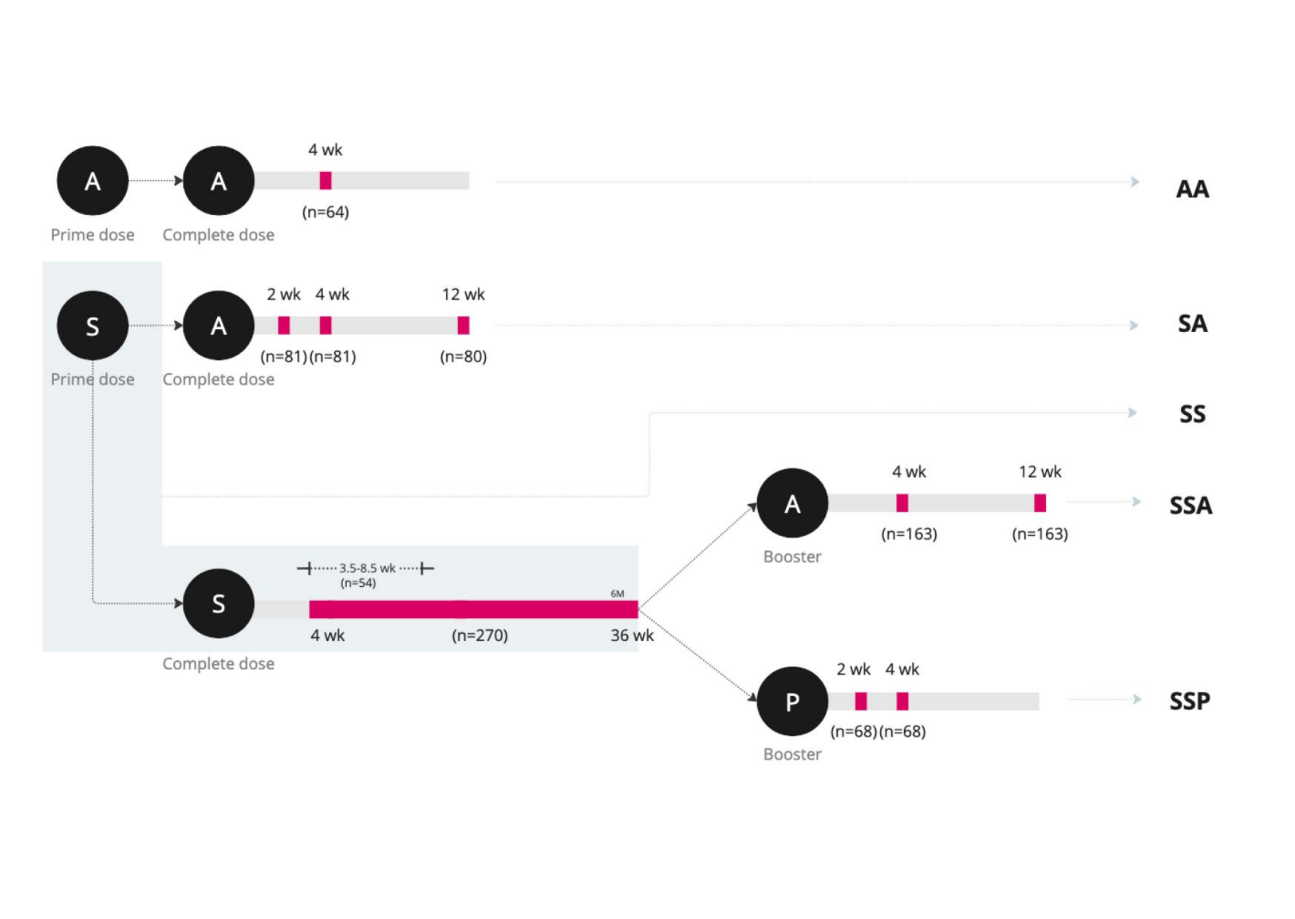
Graphical summary of sample collection across groups consisting of Sinovac CoronaVac (S), AstraZeneca ChAdOx1 nCoV-19 (A) or Pfizer BNT162b2 (P) vaccinations. For the AA group (homologous AstraZeneca), blood samples were collected 4 weeks after the second dose (n = 64). In the SA group (heterologous of Sinovac followed by AstraZeneca), samples were obtained at 2 weeks (n = 81), 4 weeks (n = 80), and 12 weeks (n = 80) post-vaccination. The SS group (homologous Sinovac) had samples collected over a range of 4 to 36 weeks (n = 270). After receiving two doses, some of SS, participants received booster doses; the SSA group (boost with AstraZeneca) had samples collected at 4 weeks (n = 163) and 12 weeks (n = 163), while the SSP group (boost with Pfizer) had samples taken at 2 weeks (n = 68) and 4 weeks (n = 68).

All participants (n = 415) were volunteers from vaccination centres linked to the Thailand Ministry of Public Health (MOPH) in Bangkok. The dataset was built through a convenience sampling strategy of healthcare professionals, whose primary role is in research and development rather than routine clinical care. Moreover, their vaccination schedule aligns with the general population, as they are not prioritised like frontline healthcare workers. Consequently, their risk of infection is comparable to that of the general public. The cohort consisted predominantly of female participants (309/415, 74.5%) with a median age of 40 years (range: 31–49 years). All participants self-reported no prior COVID-19 infection (Table 1). Participants were divided into five groups based on their homologous and heterologous prime-boost vaccination regimens: (**AA**) two doses of AstraZeneca (ChAdOx1 nCoV-19) as a homologous regimen (n = 64); (**SA**) a heterologous regimen of Sinovac (CoronaVac) followed by AstraZeneca (n = 81); (**SS**) two doses of Sinovac (n = 270); (**SSA**) two doses of Sinovac followed by a third (booster) dose of AstraZeneca (n = 163); and (**SSP**) two doses of Sinovac followed by a third (booster) dose of Pfizer (BNT162b2) (n = 68) (Fig. 1).

**Table 1:** Summary of baseline characteristic for vaccine mixing and matching study in Thailand from June to October 2021.

	Primary vaccination			Boost vaccination		Overall
	AA	SA	SS	SSA	SSP	
	N = 64	N = 81	N = 270	N = 163	N = 68	N = 415
**Gender**						
Female	51 (79.7%)	43 (53.1%)	215 (79.6%)	126 (77.3%)	59 (86.8%)	309 (74.5%)
Male	13 (20.3%)	38 (46.9%)	55 (20.4%)	37 (22.7%)	9 (13.2%)	106 (25.5%)
Age (year)*	46.0 [33.8;61.0]	43.0 [37.0;49.0]	38.0 [30.0;46.5]	42.0 [31.5;48.5]	32.0 [26.5;36.0]	40.0 [31.0;49.0]
Vaccination interval (wk)*						
Dose 1–Dose 2	11.0 [10.9;11.0]	3.00 [3.00;3.00]	3.00 [2.43;3.86]	3.00 [3.00;3.86]	3.57 [2.43;4.00]	3.0 [3.0;4.0]
Dose 2–Dose 3	N/A	N/A	N/A	9.14 [8.14;12.6]	14.4 [12.1;18.1]	11.2 [8.6;13.0]
Collection interval (wk)*						
No. time-points ** (N)	1 (64)	3 (242)	1 (270)	2 (326)	2 (135)	–
Time after 2nd Dose (wk)	4.6 [4.1;4.6]	4.0 [2.0;13.0]	12.1 [9.0;14.4]	–	–	–
Time after 3rd Dose (wk)	–	–	–	8.4 [4.0;13.0]	4.6 [2.0;4.6]	–

The table includes gender, age, and self-reported history of COVID infection. The vaccine interval is reported in weeks since previous dose. For homologous and heterologous primary vaccination, timepoints are following second vaccination. For Boost schedules, one collection followed primary vaccination with a further two after boost.

N/A—data not available; Homologous primary CoronaVac (**SS**) and ChAdOx1 nCoV-19 (**AA**). Heterologous CoronaVac, ChAdOx1 nCoV-19 (**SA**) priming and CoronaVac or ChAdOx1 nCoV-19 and BNT162b2 (**SSA** and **SSP**) boost programmes (see Fig. 1).

*Median [inter-quartile range (IQR)]

**Collected at multiple timepoints per 1 volunteer.

The interval between the two primary doses varied based on the manufacturer’s recommendations: 11 weeks for the AA group, 3 weeks for the SS group, and variable intervals for the SA group, in accordance with vaccine mixing guidelines. After completing the primary vaccination series (two doses), blood samples were collected at timepoints designed to capture the peak of antibody responses, as reported in the literature^19,20^. Specifically, sampling occurred at 4 weeks for AA, at 2, 4, and 12 weeks for SA, and between 4 and 36 weeks for SS. For participants receiving a booster dose, samples were collected at 4 and 12 weeks for SSA and at 2 and 4 weeks for SSP. Overall, the dataset consisted of 1,037 samples from 415 participants, distributed across the five vaccination groups collected from various timepoints (Fig. 1).

The age distributions across the groups were broadly similar, with median ages: AA: 46.0 years (IQR: 33.8–61.0), SA: 43.0 years (IQR: 37.0–49.0), SS: 39.0 years (IQR: 32.0–51.0), SSA: 42.0 years (IQR: 32.0–49.0), and SSP: 32.0 years (IQR: 26.5–36.0) (Table 1). However, across primary vaccine groups, there were differences (Kruskal Wallis *p* < 10^–5^)*,* with the SS group having a marginally lower median age compared to SA and AA (Wilcoxon *p* < 0.007), leading to the SSP group being the youngest (median age: SSP 32.0 vs. SSA 42.0; Wilcoxon *p* < 10^–5^), which may influence vaccine response and immune profiles. The proportion of female participants also varied by group (range: 53.1% (SA) to 86.8% (SSP)) (Table 1).

### Profiling isotype-specific responses in homologous and heterologous prime vaccination recipients

Antibody responses were profiled using a multiplex microsphere assay targeting six antigens: five SARS-CoV-2 variant-specific structural proteins and one vaccine vector-derived antigen. The assessed antigens included the Spike trimer (wildtype; WT), Receptor binding domain (RBD, WT), RBD (Delta variant), RBD (Omicron variant), Nucleoprotein (WT), and chimpanzee adenovirus (vaccine vector) (**Table S1**). Each antigen was evaluated for three antibody isotypes (IgG, IgM, and IgA), and IgG avidity was measured to assess antibody binding strength. To compare antibody profiles across different prime vaccination regimens, we selected a cohort of samples from recipients of homologous (AA, SS) and heterologous (SA) prime vaccinations. All samples were collected within the same time frame, between 3.5 and 8.5 weeks post-vaccination, as this interval provided the most overlap across the three vaccination groups. In addition, we included antibody responses for 64 negative controls who represent a pre-COVID and non-COVID vaccinated group (NEG; see **Materials and Methods**).

**Fig. 2:**
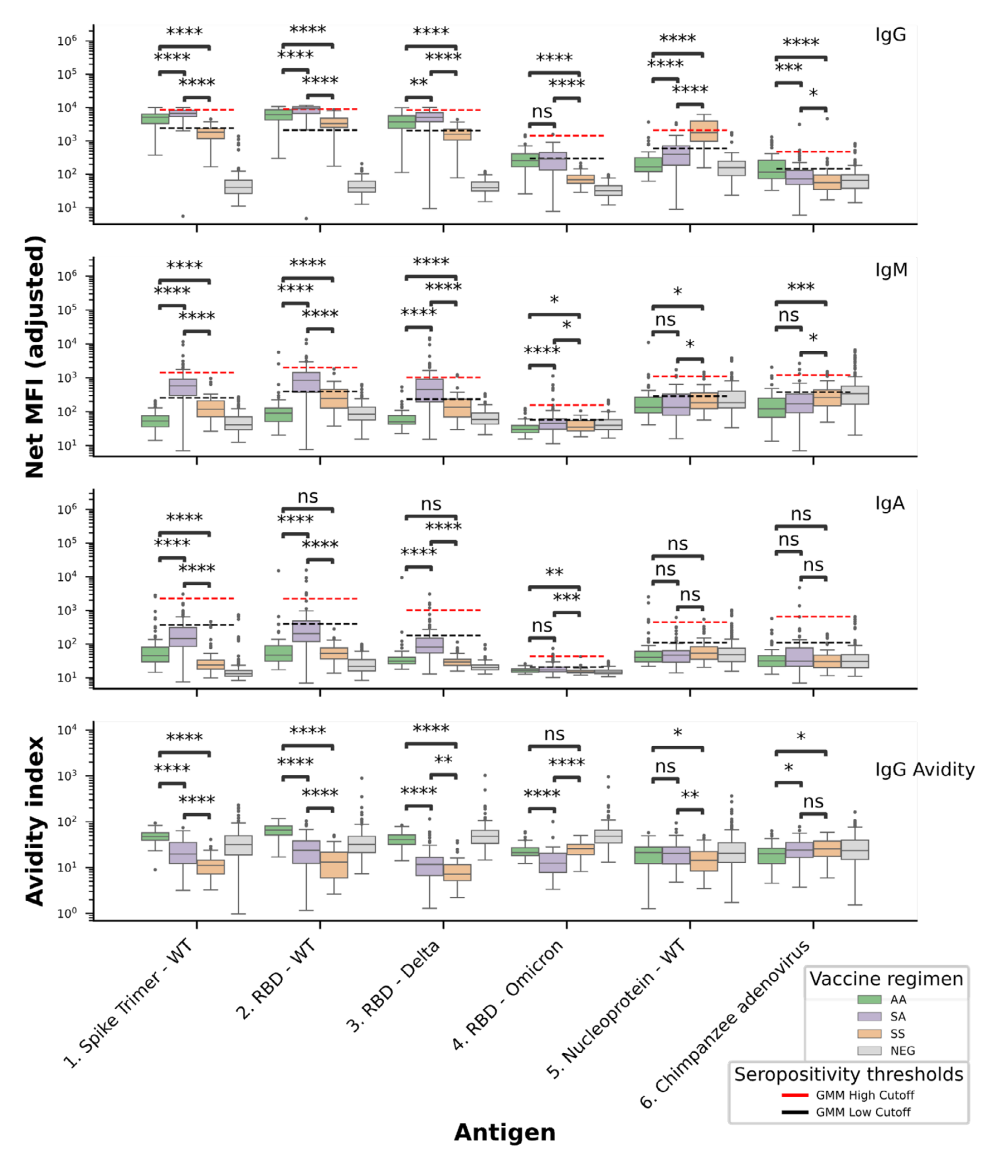
Pan-isotype profile of antibody responses to homologous and heterologous vaccination. IgG, IgM IgA and IgG avidity MFI after second homologous or heterologous vaccination with AstraZeneca ChAdOx1 nCoV-19 and ChAdOx1 nCoV-19 (AA) (n = 64), Sinovac CoronaVac and AstraZeneca ChAdOx1 nCoV-19 (SA) (n = 81), and Sinovac CoronaVac and CoronaVac (SS) (n = 270). Negative controls – unvaccinated individuals, samples collected prior to 2019 (NEG) (n = 64). Samples were collected 3.5 to 8.5 weeks after second primary vaccination. MFI was adjusted to account for batch effects. Wilcoxon signed-rank test was applied as a measure of significance (* *p* < 0.05; ** *p* < 0.01; ***p < 0.001; ****p < 0.0001). The seropositivity thresholds (high, low) are based on fitting for each antigen a Gaussian mixture model (GMM) to classify the MFI levels into three groups (negative/low, intermediate, and high).

Across all SARS-CoV-2 antigens, primary vaccination groups (AA, SA, SS) observed a significant IgG median fluorescence intensity (MFI) increase over naïve individuals (NEG) (*p* =  < 0.0001), except for one comparison (Nucleoprotein—WT: NEG 157 vs. AA 166; *p* = 0.044) (**Table S2 (IgG)**). Using GMM models applied to IgG data for each antigen (see **Materials and Methods**), we established cut-offs to classify samples into low, intermediate, and high reactivity categories (Fig. 2). We observed that most naïve samples clustered within the ‘low reactivity’ group (93%). Recipients of the heterologous (SA) regimen exhibited a significant increase in IgG, IgM and IgA levels against the trimeric SARS-CoV-2 spike antigen and the RBD WT and Delta antigens over that of the homologous (AA and SS) recipients (*p* =  < 0.0001; IgG MFI: Trimer SS:1811, AA:5174, SA:6725; WT RBD SS:3287, AA:6178, SA:9261; Delta RBD SS:1575, AA:3730, SA:9261; **Table S2**). All vaccinated groups reported significantly greater anti-Omicron RBD IgG over the naïve individuals (*p* =  < 0.0001; IgG MFI: Omicron RBD SS:68, AA:255, SA:286, NEG:31) although, responses to RBD Omicron variant were reduced significantly by at least ten-fold when compared with responses to other RBD variant antigens, despite like-for-like coupling conditions (**Table S1**). Both the AA and SA groups exhibited a significantly increased IgG response to the RBD omicron variant when compared to the SS group (*p* =  < 0.0001; median MFI: Omicron RBD SS:68, AA:255, SA:286).

As there were some potential differences in age between the primary vaccine groups, we assessed any effects on MFI levels. While no significant correlations (p < 0.05) were found between IgM or IgA MFI levels and age across all antigens, a weak negative correlation (R = −0.13, p < 0.01) was observed between IgG levels specific to SARS-CoV-2 antigens and age (**Figure S2**). An analysis using age-matched groups confirmed the same trends in antibody responses observed (results not shown). The interval time between primary and completion doses was fixed as part of the respective vaccination protocols. In addition, we fitted multivariate linear regression models on (log10) MFI with age, gender and interval time between prime and complete dose, across vaccine groups and antigens (**Table S3**). This analysis confirmed the analysis above (**Table S2**), demonstrating that recipients of the heterologous (SA) regimen showed a significant increase in IgG, IgM, and IgA levels against the trimeric SARS-CoV-2 spike antigen, as well as the RBD WT and Delta antigens, compared to recipients of the homologous (AA and SS) regimens. Furthermore, both the AA and SA groups exhibited a significantly stronger IgG response to the RBD Omicron variant relative to the SS group (**Table S3).**

### Temporal dynamics of antibody responses to heterologous boost

Participants who were previously administered the homologous SS primary regimen were vaccinated with either AstraZeneca ChAdOx1 nCoV-19 (SSA, N = 164) or Pfizer BNT162b2 (SSP, N = 68) as a heterologous boost, 36 weeks after completion of the primary regimen. Paired samples were collected at three time-points reflective of the maturation period of the vaccine response (SSA: w(eek)0, 4, and 12; SSP: w0, 2, and 4). Four weeks following heterologous boost, across both SSA and SSP regimen, an average 9.8- or 16.8-fold MFI increase in IgG MFI, respectively, was observed across all SARS-CoV-2 antigens excluding the N protein antigen (*p* =  < 0.0001; median IgG MFI: Trimer SSA w0: 1811, w4: 5174, SSP w0: 1811, w4: 5174; Omicron RBD SSA w0: 1811, w4: 5174, SSP w0: 1811, w4: 5174), an average 5.3- or 8.1- fold boost for IgA MFI (*p* =  < 0.0001; median IgA MFI: Trimer SSA w0: 25, w4: 263, SSP w0: 28, w4: 956), with a diminished IgM 1.7- or 2.0-fold boost increase observed (*p* < 0.0001; median IgG MFI: Trimer SSA w0: 85, w4: 191, SSP w0: 1811, w4: 5174; Omicron RBD SSA w0: 1811, w4: 5174; SSP w0: 1811, w4: 5174) (**Table S4**). At the 4-week time-point (w4), across the spike antigens and their derivatives, including the Delta and Omicron RBD, an average of 78% (SSA) and 98% (SSP) of samples exceeded the IgG ‘GMM high’ cut-off (Fig. 2). Notably, anti-Omicron RBD was at its highest median level recorded across the entire cohort 2 weeks after heterologous boost with BNT162b2. When compared to the SSA group at four weeks, the SSP group showed significantly greater levels  (*p* < 0.0001; median IgG Omicron RBD: SSA w4 1383, w12 613; SSP w2 3777, w4 2889). However, in both SSA and SSP regimens, anti-Omicron RBD IgG exhibited the sharpest decline (Omicron RBD IgG decline SSA: w4–w12 56%, SSP w2–w4 23%), when compared to the other spike variant antigens (average decline SSA w4–w12 13%, SSP w2–w4 0%). Similar trends were found when considering fold-changes over time using geometric means (**Table S5**).

There was a higher fold increase of IgA over the baseline (w0) when compared to IgM across all spike-derived antigens, including Omicron RBD (average fold increase: SSA IgM: 1.7, IgA: 5.3; SSP IgM: 2.5, IgA:18.2) (**Figure S3**) (**Table S4**). Across all spike antigens, both IgA and IgM began to decline after the 4-week (SSA) and 2-week (SSP) timepoints (average decline from previous timepoint; SSA IgA:70%, IgM:53%; SSP IgA:47%, IgM:84%) (**Figure S3**) (**Table S4**). At the 4-week timepoint, the SSP regimen consistently yielded a significantly greater IgA response when compared to SSA (*p* < 0.01; median IgA MFI: Trimer SSA w4:267, SSP w4:413; Delta RBD SSA w4:113 SSP w4:273; Omicron RBD SSA w4:21, SSP w4:30), while no significant difference was observed between vaccine regimen and IgM MFI (*p* < 0.05; median IgM MFI: Trimer SSA w4:191, SSP w4:173; Delta RBD SSA w4:163 SSP w4:111; Omicron RBD SSA w4:41, SSP w4:40) (**Figure S3**) (**Table S4**). To confirm the results at week 4, we fitted multivariate linear regression models on (log10) MFI with age, gender and interval time, and vaccine booster groups (SSP vs. SSA), across antigens (**Table S6**). This analysis confirmed that the higher MFI observed in the SSP groups for certain antigens across IgA and IgG isotypes, as well as the broader variations in antibody levels, are primarily driven by the differences in vaccine regimens rather than demographic factors such as age or gender among the groups.

### Anti-SARS-CoV-2 IgG avidity in homologous/heterologous prime and boost recipients

IgG avidity was measured as a part of the Luminex multiplex assay. The avidity index metric represents the MFI of IgG that remains bound to the antigen following dissociation of weakly bound immunoglobulins. Therefore, the naive pre-COVID control samples (NEG) were removed from the analysis as any, albeit infrequent, non-specific interactions would yield incoherent avidity indices. IgG avidity responses to the trimeric SARS-CoV-2 spike antigen, as well as the RBD wild-type (WT-RBD) and Delta variants, were significantly higher in the homologous AA regimen compared to the homologous SS group. The SA group exhibited intermediate avidity, higher than SS and lower than AA (*p* < 0.001; median avidity indices: Trimer SS: 11, AA: 47, SA: 19; WT RBD SS: 13, AA:66, SA:23 and Delta RBD SS:7, AA:40, SA:11). Interestingly, the SS regimen exhibited a significantly greater Omicron RBD avidity index over that of the SA regimen (*p* < 0.0001; median avidity indices: Omicron RBD SS:25, SA:12), with no significant difference in IgG avidity between SS and AA groups (*p* > 0.05; median avidity indices: Omicron RBD SS: 25, AA: 21).

Across heterologous boost recipients, a consistent pattern of significant IgG avidity index decline after the second timepoint (w2) was observed (*p* < 0.0001; median avidity indices: Trimer SSA w4:90, w12:55; SSP w2:118, w4:102; WT-RBD SSA w4:114, w12:71; SSP w2:123, w4:108; Delta RBD SSA w4:70 w12:71; SSP w2:115, w4:94) (**Figure S3**) (**Table S5**). Omicron RBD responses were not consistent with this pattern, exhibiting instead, a decline across all three respective timepoints, more pronounced in the SSA group than SSP (*p* < 0.05; median avidity indices: Omicron RBD SSA w0: 28: w4:22, w12:18; SSP w0:28: w2:28, w4:24) (**Figure S3**) (**Table S4**). Anti-Omicron IgG bore the greatest avidity of any analyte at the w0 timepoint, reflecting the significant increase in homologous prime avidity observed previously (Fig. 1**)** (**Table S4**).

### Longevity of antibody responses to inactivated vaccine nucleoprotein antigen

**Fig. 3:**
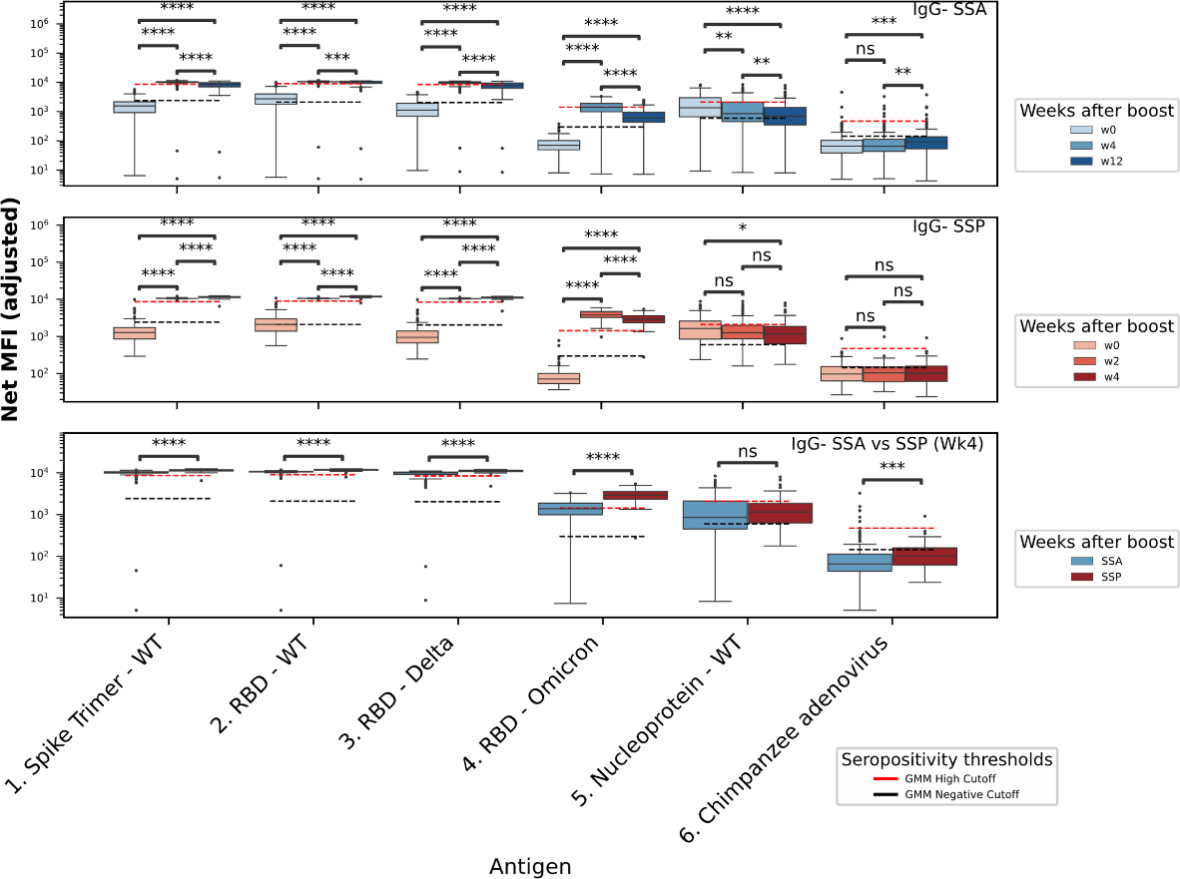
IgG significantly increases in homologous CoronaVac recipients after heterologous boost with ChAdOx1 nCoV-19 and BNT162b2. The IgG panel against the Spike trimer, receptor-binding domain (RBD), nucleoprotein and chimpanzee adenovirus are displayed in both SSA (n = 163) (top) and SSP (n = 68) (middle) regimens together with the level of antibodies in both groups at 4 weeks (bottom). *p < 0.05; **p < 0.01; ***p < 0.001; ****p < 0.0001. The seropositivity thresholds (high, negative) were determined by fitting a Gaussian mixture model (GMM) to the MFI levels for each antigen, classifying them into three groups (negative/low, intermediate, and high).

SARS-CoV-2 nucleoprotein (N) seroconversion occurs when recipients are vaccinated with an inactivated whole-virus platform, or natural infection. All participants were screened for anti-N IgG, IgM and IgA antibodies and for IgG avidity. IgG responses were significantly greater in regimens featuring the CoronaVac whole inactivated virus platform, SS and SA (*p* < 0.0001; median MFI: Nucleoprotein SS:1767, AA:166, SA:398) (Fig. 2). Despite some weakly significant indifferences, anti-N IgM indicated no increase over the naïve negative (NEG) population (n = 64; *p* < 0.05; IgM median MFI: Nucleoprotein SS:183, AA:135, SA:134, NEG:184) as was the case for IgA (*p* > 0.05; IgA median MFI: Nucleoprotein SS:53, AA:40, SA:46, NEG:48). Across the 12-week sampling period for the SSA heterologous boost population, a significant decline of anti-N IgG was observed (*p* =  < 0.0001; IgG median MFI: Nucleoprotein SSA w0:1350, w4:855 w12:691). In the SSP group, a less pronounced but still significant decline was observed over four weeks  (*p* < 0.05; IgG median MFI: Nucleoprotein SSP w0:1631, w4:1155). No significant difference was observed for anti-N IgG in SSP/SSA at the 4-week timepoint (*p* > 0.05; IgG Median MFI: Nucleoprotein SSA w4:855, SSP w4:1155) (Fig. 3).

The analysis of anti-N IgG levels in CoronaVac recipients (double dose, SS group, n = 730) over a 28-week window post vaccination revealed a waning response. Seroconversion rates decrease from 84% at 8 weeks to 60% at 16 weeks and further to 49% at 24 weeks, with over 66% below the ‘low reactivity’ threshold after 28 weeks. In the vaccine mixing group with a single dose of CoronaVac and ChAdOx1 nCoV-19 (SA group, n = 241), seroconversion starts at 28% at week 8 and decreases over the 28-week period). Linear regression analysis of the anti-N IgG MFI level as a function of time since the last CoronaVac vaccination (**Figure S4**) indicates a steady decrease in anti-N levels over time for both SS and SA groups (SS: correlation efficient R = -0.25, *p* < 0.0005; SA: R = -0.13, *p* = 0.037).

### Profiling antibody responses to Y25 Chimpanzee adenovirus vector recipient

A synthetic construct, coding for a truncated portion of the Y25 Chimpanzee adenovirus surface hexon protein, was included in the panel to detect host responses to the ChAdOx vector. IgG responses were significantly higher in participants vaccinated with the ChAdOx vector (AA and SA) (*p* < 0.001; median MFI: Chimpanzee adenovirus SS:55, AA:115, SA:72). However, this trend was not observed for IgM and IgA isotypes. A significant increase in anti-hexon IgG and IgA levels was observed from week 0 to week 12 in the SSA (ChAdOx) heterologous boost cohort (*p* < 0.001; IgG median MFI: Chimpanzee adenovirus (hexon) SSA w0: 65, w12: 90). In contrast,  no such increase was detected in the SSP (BNT162b2) regimen (*p* > 0.05).

### Modelling antigen responses to characterise immunised and infected populations

In this study, we incorporated antibody data from an additional cohort of vaccine breakthrough infections (“Positive”, n = 281). To support serological surveillance within the context of inactivated vaccination platforms, we leveraged multi-isotype antibody profile data (log10 MFI) and applied Gaussian Mixture Models (GMMs) to cluster individuals into 4 or 5 distinct groups (“components”). These clusters were then compared against five predefined groups: Pre-COVID (NEG), AA/SA, SS, Vaccine Breakthrough (Positive), and Booster SSA/SSP. For certain analyses, the Positive and Booster groups were merged, resulting in four primary categories. An iterative approach was employed to optimise the GMMs, identifying the most informative antigen-isotype feature combinations (2-, 3-, or 4-dimensional) for distinguishing the 4 or 5 components or clusters (**Table S7**).

**Fig. 4:**
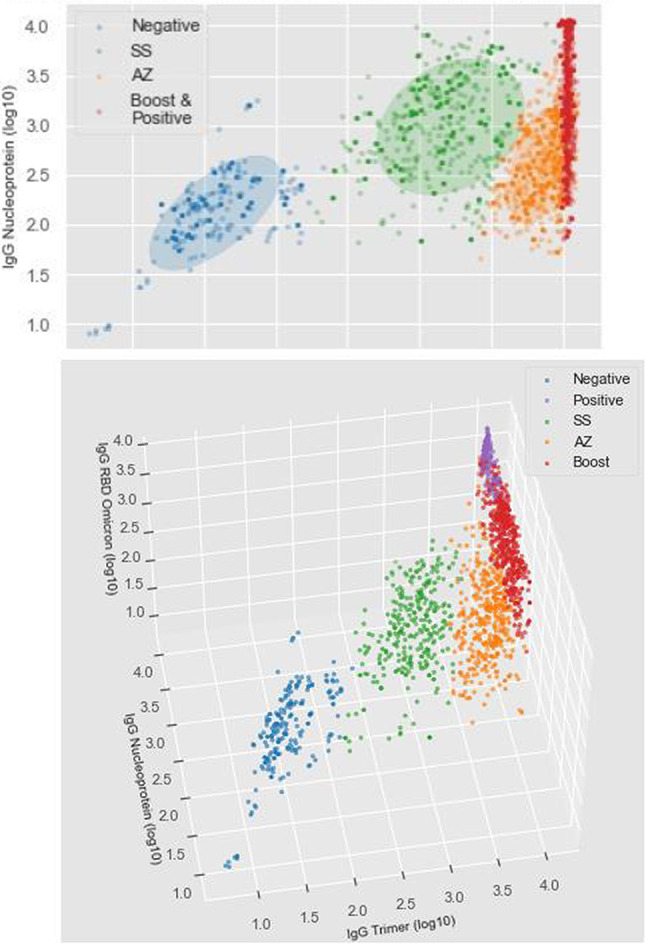
Finite Gaussian mixture modelling (GMM) to identify clusters (components) using combinations of antigen and isotype responses (dimensions), with the overlayed colours indicating actual vaccine groups (Negative/Pre-COVID (n = 64); SS (n = 270); AZ = SA (n = 81) or AA (n = 64); Positive = convalescent / vaccine breakthrough (n = 281); Boost = SSA (n = 163) or SSP (n = 68). (Top) 2D model (WT SARS-CoV-2 spike timer, WT nucleoprotein IgG) with 4 components (F-score = 0.88); the ellipses are the estimated covariances, centred on the mean for each component. (Bottom) 3D model (WT SARS-CoV-2 spike timer, WT nucleoprotein IgG, RBD Omicron IgG) with five components (F-score = 0.82).

A two-dimensional GMM model incorporating the Spike Trimer (IgG) and Nucleoprotein antigen (IgG) achieved the highest precision and recall, with an overall F-score of 0.88 (**Table S7; **Fig. 4). The Pre-COVID group (NEG, n = 64) was classified with perfect accuracy (F-score = 1), while the homologous CoronaVac group (SS, n = 270) also showed high accuracy (F-score = 0.93). In contrast, the homologous ChAdOx1 nCoV-19 primary recipient group (SA/AA) had lower classification performance (F-score = 0.71), as did the positive/booster groups (F-score = 0.86). Misclassifications were most frequent between the positive and booster groups, with 34 out of 42 cases (81.0%) incorrectly assigned. Extending the GMM model to include a fifth component (number of Gaussian components = 5) did not improve overall accuracy (F-score = 0.78; **Table S7**). However, adding a third feature—RBD Omicron IgG—enhanced the F-score of the five-component model by approximately 5% (F-score = 0.82; Fig. 4**, Table S7**). Despite this improvement, the accuracy for the booster (F-score = 0.70) and positive (F-score = 0.70) groups remained low. Overall, the GMM models indicate that the four-component approach provides better predictive accuracy than models with five clusters (**Table S7).**

## Discussion

The application of heterologous SARS-CoV-2 vaccination during the COVID-19 pandemic was initially driven by necessity, but outcomes have proven favourable, with numerous peer-reviewed studies validating their use and efficacy. In our study, we profiled a cohort of recipients of both homologous and heterologous primary and booster vaccines, examining the diversity and strength of antibody responses across inactivated, vectored, and mRNA platforms. Additionally, paired analyses investigated the dynamics of different heterologous booster regimens. We developed and tested a robust model that accurately classifies immune responses and infers vaccination and infection history using only three IgG metrics per sample.

The cohort represents a convenience sample of healthcare staff involved in research and development roles, whose vaccination schedules align with those of the general population. Their risk of infection is likely comparable to that of the broader public. Analyses grouping the cohort by three homologous and heterologous primary vaccine regimens indicated the heterologous CoronaVac/ChAdOx1 nCoV-19 (SA) schedule yielded significantly stronger IgG, IgM and IgA responses when compared to homologous recipients (SS and AA). High levels of anti-SARS-CoV-2 antibodies and their avidity across IgG, IgM and IgA enhance protective immunity. High immunoglobulin avidity indicates affinity maturation and effective neutralisation, highlighting the critical role of isotype interplay and avidity maturation in establishing strong immune defence^25,26^. A review of 48 SARS-CoV-2 vaccination studies, 12 of which covered schedules with heterologous inactivated/vectored platforms, found that in all cases CoronaVac/ChAdOx1 nCoV-19 heterologous primary vaccination resulted in an average 5.6-fold increase in antibody response over that of homologous inactivated regimens, with no coherent trend observed in the six examples of heterologous priming as opposed to homologous vectored regimens (i.e. AA vs. SA)^9^. Our analyses indicate that the heterologous CoronaVac/ChAdOx1 regimen outperformed the homologous regimens, generating significantly higher levels of IgG, IgA and IgM against WT and Delta spike antigens. Research scrutinising the nature of enhanced heterologous inactivated/vector priming in mice found higher antibody and cell-mediated immune responses compared to a homologous vaccination, driven by enhanced innate activation, resulting in a more mature memory and plasma B-cell response. Moreover, moderate IgA enhancement was noted in heterologous recipients, which was observed here through a 2.5- to 4.3-fold increase in IgA over homologous regimens^27^. Previous studies have detected antibodies against chimpanzee adenovirus in human sera from sub-Saharan Africa, the United States, and Thailand^28^. Further, the Hexon antigen of chimpanzee adenovirus shares genetic similarity with human adenovirus species HuAds, ChAd24, and RhAd53, potentially leading to cross-reactivity in antibody tests between these viruses^29^.

Reactivity to the Omicron RBD antigen after priming and heterologous boost with vaccines designed against the wild-type variant of SARS-CoV-2 was reduced significantly across all analytes when compared to the RBD WT and Delta variant antigens, consistent with previous studies^30,31^. The increase in anti-Omicron IgG after boosting has been described previously^32. However^, the lack of IgM or IgA response when compared to both WT and Delta highlight the effects of Omicron evasion. Unlike the overall yield of IgG, IgA and IgM, the avidity of the heterologous IgG response was reduced when compared to the homologous vectored AA group. However, the homologous inactivated SS group yielded the responses with the lowest avidity indices. Although homologous vaccination might be expected to produce a matured response through consistent B-cell activation, this does not appear to hold true for the CoronaVac vaccine. Contrary to this expectation, previous reports have shown greater avidity in SS vaccination compared to homologous BNT162b2^33^ regimens. Addtionally, heterologous ChAdOx1/BNT162b2 regimens have demonstrated higher avidity responses than homologous BNT162b2/BNT162b2^34,35^ regimens.

The detection of anti-RBD levels and their avidity correlates strongly with the neutralizing capacity of the immune response against SARS-CoV-2. A systematic review and meta-analysis indicated that heterologous vaccination regimens, particularly combinations of CoronaVac (CV) and ChAdOx1 (ChAd), yield greater anti-RBD IgG and neutralizing antibodies against wild-type and delta variants compared to homologous regimens of either vaccine alone^29^. Our study aligns with this, having observed higher RBD antibody levels and avidity in the heterologous prime vaccination group (SA group), suggesting a more robust immune response compared to homologous approaches. Additionally, studies on homologous and heterologous booster doses—using BNT162b2 (Pfizer-BioNTech), mRNA-1273 (Moderna), or Ad26.COV2.S (Johnson & Johnson-Janssen)—show significant increases in binding antibodies and neutralizing antibodies against SARS-CoV-2 pseudoviruses^36^. The increases in antibody levels following heterologous boosting are generally comparable to or greater than those after homologous boosting^36^, highlighting the enhanced immunogenicity associated with both booster vaccinations and heterologous vaccination strategies, suggesting they may confer additional protective benefits against SARS-CoV-2.

Across heterologous boost groups, paired samples were collected over a 4- and 12-week period. A comparison at the 4-week time point indicated that the SSP group (CoronaVac/CoronaVac/BNT162b2) had a significantly greater IgG and IgA response to all SARS-CoV-2 antigens, including the Omicron RBD, over that of the SSA group (CoronaVac/CoronaVac/ChAdOx1). A similar study in Bangkok on healthcare workers presented comparable findings, noting also, that SSA antibodies demonstrated a steeper decline after 12 days^37^. Host immune responses to viral-vectored vaccine platforms have been extensively studied, particularly in the context of human adenoviral vectors^38^. Memory responses elicited by natural adenovirus infections can hinder payload delivery through vector neutralisation and cytotoxicity, reducing vaccine efficacy^39^. Our observation of lower antibody responses to SARS-CoV-2 in viral-vectored vaccine platforms compared to mRNA vaccine platforms highlights a notable difference in antibody responses between the two vaccine platforms, implying a potential variation in vaccine efficacy.

Understanding the dynamics of anti-N IgG waning, specifically in the context of inactivated vaccine platform recipients, is key to applying sero-epidemiological tools in SARS-CoV-2 infection control. Numerous examples of studies leveraging anti-N responses in differentiating vaccinated individuals from natural or breakthrough infections are present in the literature^40,41^. However, the same anti-N antibodies indicative of infection are also reared against components of inactivated platforms, which confound these inferences. Our findings demonstrate that, while heterologous primary immunisation with a CoronaVac/ChAdOx1 rears a diminished response, a homologous CoronaVac/ CoronaVac schedule yields a strong anti-N response, comparable to that of natural or breakthrough infection, which takes ~ 20 weeks to wane.

Developing a robust method to distinguish host vaccine responses from infection in high-transmission settings with heterologous vaccination regimens is crucial for SARS-CoV-2 surveillance. Such a tool could complement serosurveys of the general population or biobank samples, providing valuable insights into high-risk communities (e.g., care homes) and broader immunisation patterns. This approach would enable a deeper understanding of transmission dynamics and host immunity, guiding strategic decisions around booster administration and control measures. Our preliminary model, which utilises a combination of three analytes—IgG against wild-type Spike trimer, nucleoprotein, and Omicron RBD—shows promise in clustering responses to accurately classify individuals as negative/unvaccinated, inactivated vaccine recipients, vectored vaccine recipients, boosted vaccine recipients, or convalescent individuals. With these classifications, and further refinements as more data become available, this approach could offer more detailed assessments of population immunity than commercial SARS-CoV-2 serology platforms currently allow. The model could be enhanced by incorporating additional features, such as convalescence duration, breakthrough infection and vaccination history, or host factors like age, to improve predictive accuracy. Nevertheless, even in its current form, the model illustrates how multidimensional serological data can enhance analytical capabilities and deepen our understanding of immunity and transmission. The most frequent misclassifications occurred between the positive/boost and ChAdOx1 primary/boost clusters, reflecting the common heterogeneity in host responses to infections of varying severities^42,43^. Breakthrough or natural infections often elicit antibody responses similar to those observed in vaccinated individuals. Depending on the application, the more accurate four-component model may be preferable when distinguishing between booster and natural infection responses is unnecessary. Although more complex classification methods, such as XGBoost models with additional features, were evaluated, they produced similar results to the GMM-based approach. The GMM, which required only three analytes, demonstrated comparable performance while remaining simpler to implement. This highlights the potential of straightforward yet effective models, though further methodological research in this area is ongoing.

Overall, our results demonstrate the advantages of implementing a heterologous regimen, specifically using CoronaVac/ChAdOx1 nCoV-19 schedules. We observed a significant enhancement in antibody levels with a heterologous boost administered four weeks post-initial vaccination. Additionally, our data successfully discriminated between breakthrough infections and various vaccination types. This facilitated the development of a model that can support future serological surveillance and provide valuable insights into virus circulation dynamics in environments with multiple vaccination regimens^44^.

## Materials and methods

### Vaccine mixing and matching cohort

A cohort of 415 healthy individuals was randomly selected from vaccination centres across the Thai Ministry of Public Health (MOPH) during the vaccine programme rollout. Whole blood samples were collected across four months from 29/06/21 to 23/10/21 throughout vaccine deployment (**Figure S1**). The timing of sample collection across the 5 groups was informed by manufacturer’s guidance: (i) homologous primary CoronaVac (SS; n = 270, 3–18 weeks), homologous ChAdOx1 nCoV-19 (AA; n = 64, 2–5 weeks), and heterologous CoronaVac and ChAdOx1 nCoV-19 (SA; n = 81, 2–14 weeks). Groups receiving a boost after homologous CoronaVac had collections at three intervals: (iv) CoronaVac/CoronaVac + ChAdOx1 nCoV-19 (SSA, n = 164, 0, 4 and 12 weeks) and (v) CoronaVac/CoronaVac + BNT162b2 (SSP, n = 68, 0, 2 and 4 weeks) (Fig. 1). Whole blood specimens were collected from each individual and stored in EDTA containers. Plasma was obtained by centrifugation at 3000 × g for 10 minutes at room temperature. Prior to serological testing, all plasma samples were aliquoted into cryogenic vials and stored at −80 °C to prevent multiple freeze–thaw cycles. Participants were surveyed at the time of collection, yielding metadata for each sample, which included age, gender, and history of vaccination and COVID-19. The study was approved by the research ethics committee, Department of Medical Sciences, Thailand Ministry of Public Health (EC15/2564). All methods were performed in accordance with the relevant guidelines and regulations. Informed consent was obtained from all subjects involved in the study.

### Pre-COVID and vaccine breakthrough cohorts

A breakthrough infection modelling cohort comprised a randomly selected group of 281 individuals who experienced a 4-week convalescent period. These individuals were specifically identified as part of a Thailand MOPH study of vaccine breakthrough infection, having been vaccinated but subsequently infected with the Omicron strain of the virus between January 2022 and March 2022. Following a confirmed positive result through RT-PCR, samples were collected four weeks post-infection. These are also referred to as a convalescent group. Further, we had blood samples and data from 44 female healthy controls collected as part of a Thailand MOPH pregnancy and disease study in 2016, as well as from 20 healthy female and male MOPH staff in 2018. These samples (n = 64) were confirmed as COVID negative via ELISA, and used as an internal controls and represent a pre-COVID and non-COVID vaccinated group. Whole blood specimens were gathered from each participant with the same procedure as mentioned above.

### Expression of Y25 recombinant hexon protein

The amino acid sequence for the Y25 chimpanzee adenovirus hexon protein was sourced from GenBank (Accession: AEW43620.1) and analysed for sequence identity with human-infecting adenovirus species. The amino acid sequence was re-codonised for expression in *Escherichia coli* and synthesised as a dsDNA construct. The Y25 chimpanzee adenovirus antigen [amino acids 129–313] was expressed as a GST fusion in a pGEX-4T-2 vector and affinity purified using glutathione resin (Thermo: PI25236) as per the manufacturer’s instructions.

### Antigen coupling

Six recombinant antigens were coupled to respective Luminex MagPlex® beads (Luminex Corporation: MC100XX), as described previously^45^. Briefly, all magnetic beads were first treated in an N-hydroxysulfosuccinimide (NHS) (Thermo: 24510) and 1-ethyl-3-(3-dimethylaminopropyl) carbodiimide hydrochloride (Thermo: 77149) activation solution. Coupling concentrations were determined by calculating a binding curve EC50 following a titration against pooled immune sera for respective antigens. Antigen orthologues were coupled at the same concentrations to permit direct comparison (**Table S1**). The coupled beads were counted for quality control using a haemocytometer and their concentration normalised. The antigen panel (**Table S1**) was chosen to capture a range of SARS-CoV-2 responses, including trimeric and receptor-binding domain (RBD) antigens to specific variants (Wuhan, Delta, and Omicron) with the addition of a Y25 chimpanzee adenovirus hexon antigen to capture potential responses to the adenovirus vaccine vector. The SARS-CoV-2 structural protein antigens include the RBD (Receptor Binding Protein), a full trimeric spike with stabilising mutations, and a wild-type nucleocapsid (N) protein.

### Luminex assay procedure

Titration, coupling and multiplex analysis of serological samples was performed as described previously^46^. In 798 µl of Buffer B (**Table S8**), 2 µl of each sample were diluted at 1 in 400 dilution ratios, a concentration previously determined to fit the dynamic range of the assay. Similarly, positive and negative control sera were used, including convalescent plasma (NIBSC 20/B5570, NIBSC, United Kingdom) from positive individuals, samples from Thai individuals within the cohort who tested positive, tand a negative panel comprising 64 blood samples from healthy individuals in Thailand collected prior to the COVID-19 pandemic. All specimens were aliquoted and stored at −80 °C until use. When preparing beads for detection, all coupled beads were pooled and mixed thoroughly in Buffer A. A total of 50 µl per bead was added into each well of a 96 well flat bottom plate (Bio-Plex Pro, Biorad 171025001) before magnetising and washing with 100 µl PBST. A total of 50 µl of each sample or control sera was used. Plates were shaken at 600 RPM for 90 minutes at room temperature in the dark. Following incubation, a 100 µL washing step was repeated three times and 50 µl of PE-conjugated anti-human IgG, IgM or IgA antibody (BioLegend, Switzerland), diluted in buffer A at a 1 in 5000 ratio, was added and incubated for 90 minutes with shaking at 600 RPM at room temperature in the dark. Plates were washed three times. Then, 50 µl buffer A was added and incubated at 600 RPM in the dark for 30 minutes at room temperature. After washing with 100 µl PBS for three times, the 96 well plates were read on the Luminex 200 bioanalyser.

#### Antigen panel and assay setup

To profile IgG, IgA and IgM antibody responses to SARS-CoV-2 immunisation and pre-existing responses, a multiplex microsphere assay panel was constructed, consisting of SARS-CoV-2 RBD (Wuhan-Hu-1 (WT-RBD), B.1.617.2 (Delta) and B.1.1.529 (Omicron) antigens, Nucleoprotein (WT-N) and a stabilised trimeric Spike (WT-Spike). The antigens were manufactured by the Native Antigen Company. Finally, a novel recombinant Y25 chimpanzee hexon protein antigen (purity 70%) was incorporated into the assay panel to detect antibodies reared against the ChAdOx1 nCoV-19 vaccine vector. In addition to the three isotypes screened, a further IgG avidity assay was performed. The IgG avidity assay was performed in parallel with the standard IgG assay. Samples were plated in duplicate wells for both IgG and IgG avidity measurements. After the initial incubation with the sample, an additional step was introduced for the IgG avidity assay. Specifically, following sample incubation, 50 µL of 2 M guanidine hydrochloride (GuHCl) was added to one set of wells and incubated for 15 minutes at room temperature to disrupt weak antibody-antigen binding. After incubation, wells were washed with PBST, and the PE-conjugated anti-human IgG antibody (BioLegend) was added as described above. The IgG avidity was calculated by determining the ratio of mean fluorescence intensity (MFI) in the GuHCl-treated wells to the MFI of untreated IgG wells, providing a measure of antibody binding strength.

### Data processing and analysis

For the quality control of Luminex data, Levey-Jennings analysis was performed, derived from the half maximal effective concentration (EC50) obtained from a 4-parameter logistic model built on the titration of positive control standards on every plate. Each plate was assessed for inter-batch variability. Any plate outside of a two-standard deviation (SD) threshold was repeated. The minimum bead count for a sample to pass quality control was 30. All mean fluorescence intensity (MFI) measurements with a bead count less than 30 were excluded from the analysis. The EC50 for each analyte was used to normalise the MFI measurements of each antigen across all plates. Metadata for each sample was combined with the adjusted MFI values for each isotype and the avidity index data using R statistical software (4.2.0)^47^. Plots were constructed using R ggplot2 (3.4.2)^48^, Python matplotlib^49^, and Seaborn^50^.

#### Gaussian mixture modelling of vaccine responses

After quality control, Gaussian mixture models (GMMs) were constructed using sklearn software^51^ to identify the most informative data points for predicting vaccine regimens. These models were built for each antigen and isotype, including avidity indices. For each combination of antigen and isotype, this process led to two MFI thresholds (negative/low, high) allowing the partitioning of values into negative/low, medium/intermediate, and high reactivity groups (**Figure S5**).

Gradient boosted trees (GBTs) were implemented on the MFI data to predict five groups: pre-COVID (“Negative”, n = 64), convalescent individuals (“Positive”, n = 281), AstraZeneca (AZ) ChAdOx1 primary group (AA/SA; n = 145), Sinovac CoronaVac (SS) primary (n = 270), and those with AZ/Pfizer booster vaccines (SSP/SSA; n = 231). The implementation of GBTs was carried out using the XGBClassifier tool^51^, and allowed for the ranking of the importance of the isotype and antigen combinations, leading to the identification of the Spike Trimer WT (IgG), RBD Omicron (IgG), RBD WT (IgG Avidity), Spike Trimer WT (IgG Avidity), RBD Delta (IgM), RBD Delta (IgM), and Nucleoprotein WT (IgG) combinations as being the strongest predictors (all importance statistics > 0.04). This set of 7 variables is consistent with other analyses presented. GMM models were then fitted to combinations of these seven these variables (or features), specifying models consisting of 3 to 6 components (or clusters to be defined, each with an estimated mean and covariance). The configurations were evaluated using F-score calculations, along with the Akaike information criterion (AIC) and the Bayesian information criterion (BIC). The highest scoring models were then built and trained using an 80:20 training to test split. Comparison of an individual’s allocated GMM cluster with their actual group (e.g., Negative, AA/SA) allowed for an estimation of misclassification errors.

## Electronic supplementary material

Below is the link to the electronic supplementary material.

Supplementary Material 1

## Data Availability

The raw mean fluorescence intensity (MFI) data of each sample is available. All scripts for data analysis are available on GitHub (https://github.com/dan-ward-bio/COVID_immunology).
