# Supplementary material for: Serological insights from SARS-CoV-2 heterologous prime and boost responses in Thailand
Source: Sci Rep. 2025 Jan 9;15:1519. doi: 10.1038/s41598-024-84392-2 (PMC11718049; doi:10.1038/s41598-024-84392-2)
Supplement: Supplementary file 1 — Supplementary Material 1 [file 41598_2024_84392_MOESM1_ESM.pdf]

Supplementary materials

Supplementary figures

Figure S1. Timeline of immunisation programs in Thailand

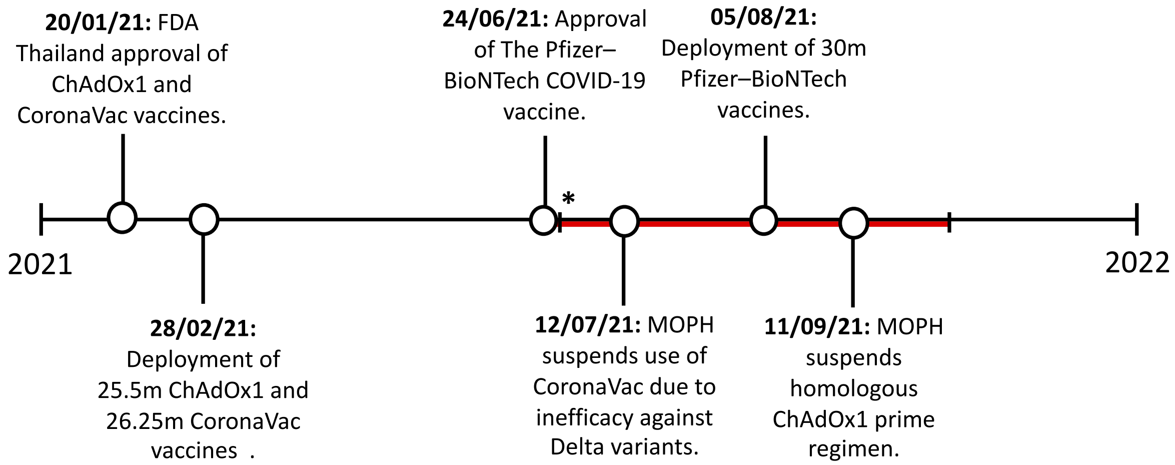

***** 23/10/21: Sample collection start; 29/06/21: Sample collection end.

Figure S2. Correlations between age and IgG, IgA, IgM, and IgG avidity levels across SARS-CoV-2 antigens. The scatter plots show the correlations between age and antibody levels (measured as median fluorescence intensity, MFI) for the 415 participants across different isotypes - (A) IgG and IgG avidity, (B) IgA and IgM — across five SARS-CoV-2 antigens. Data were generated from samples taken 3.5 to 8.5 weeks after prime vaccination in recipients of homologous (AA, SS) and heterologous (SA) vaccination regimens. The Spearman's correlation coefficients (R values) and p-values are displayed for each comparison, indicating the strength and significance of the relationship between age and antibody levels.

(A) IgG (top) and IgG avidity (bottom)

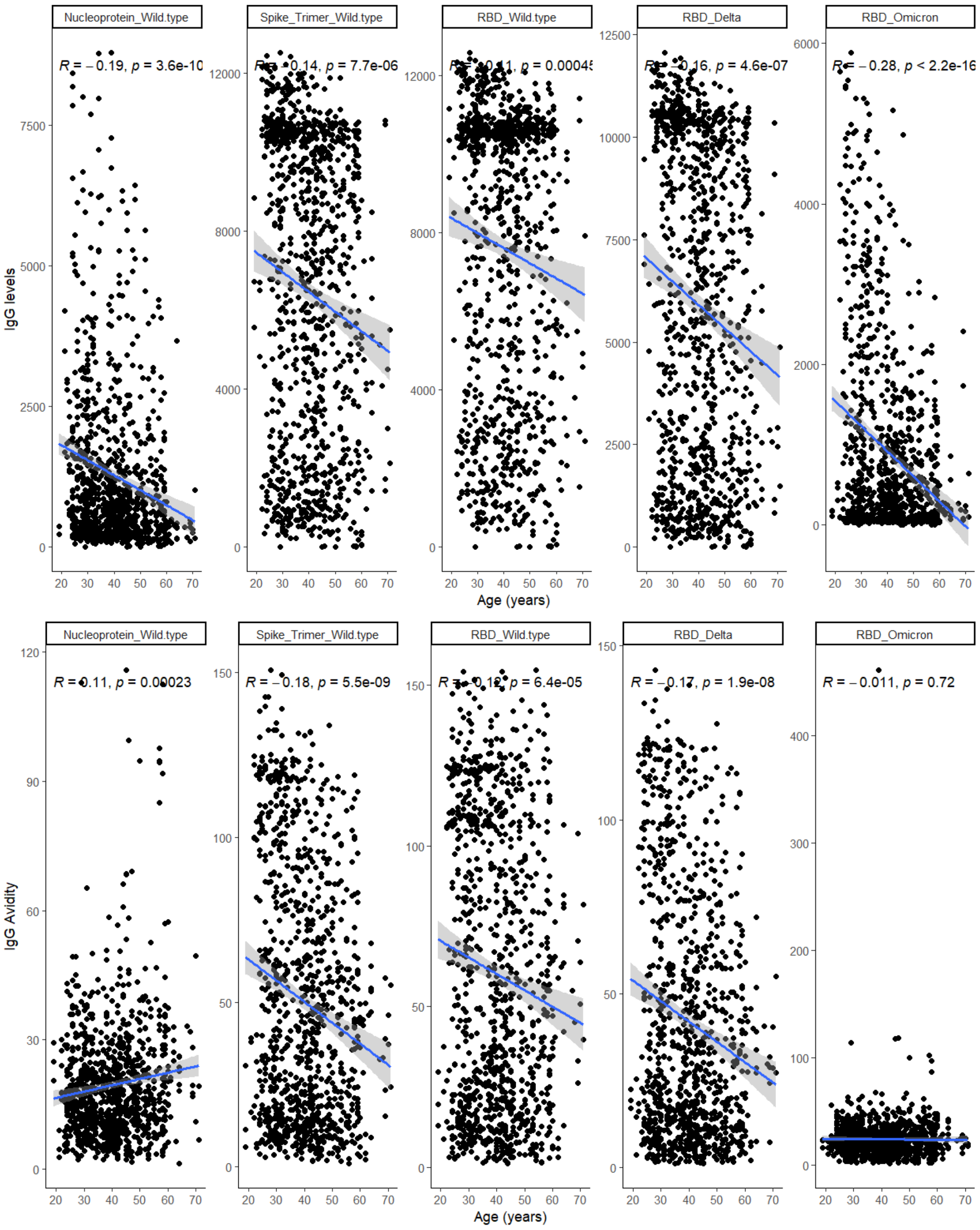

(B) IgM (top) and IgA (bottom)

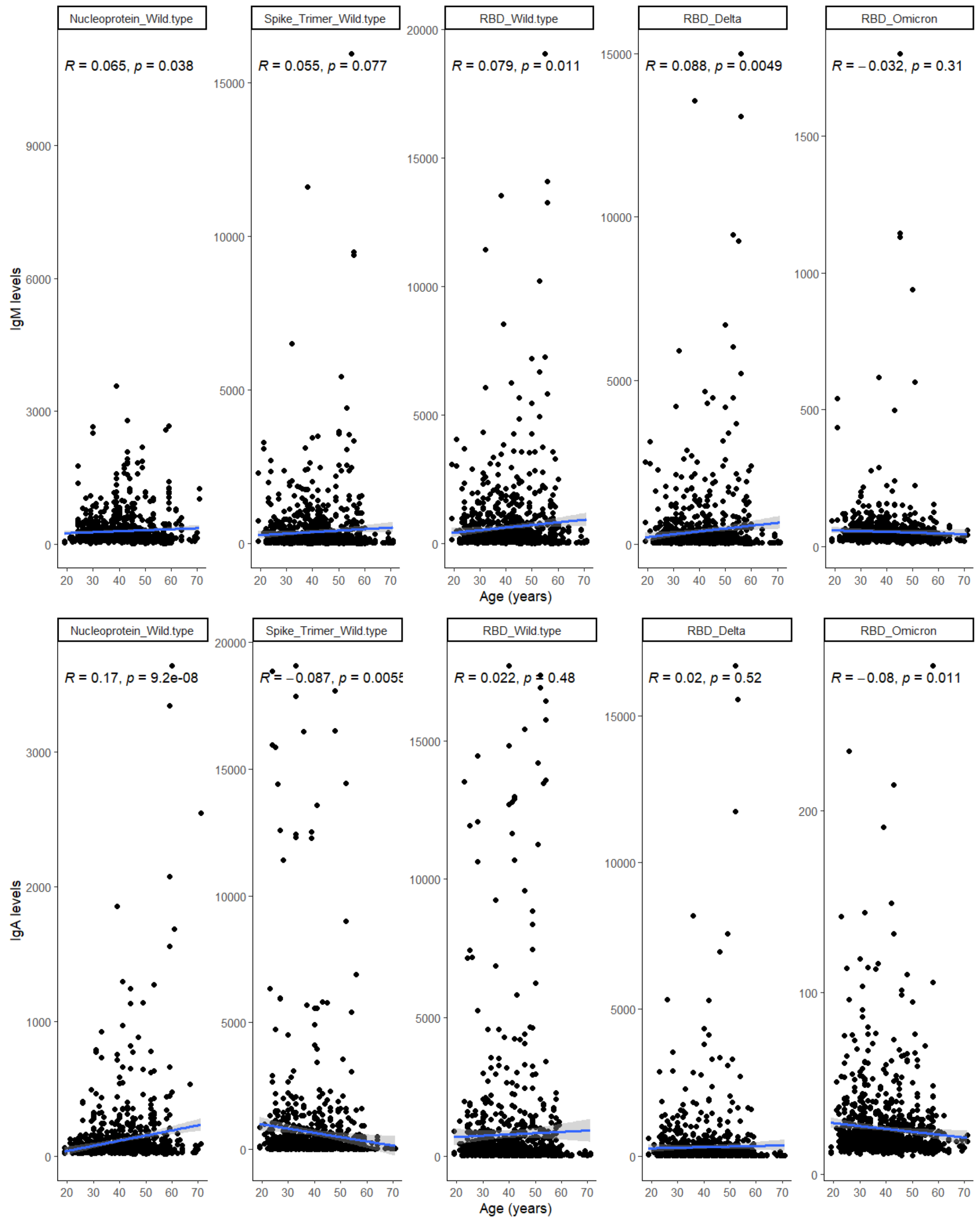

Figure S3. IgM and IgA and IgG avidity responses to heterologous boost vaccination across three time intervals following vaccination. Note, Figure 3 shows the results for IgG responses

(A) IgM (SSA top; SSP middle; SSA vs. SSP (at week 4) bottom)

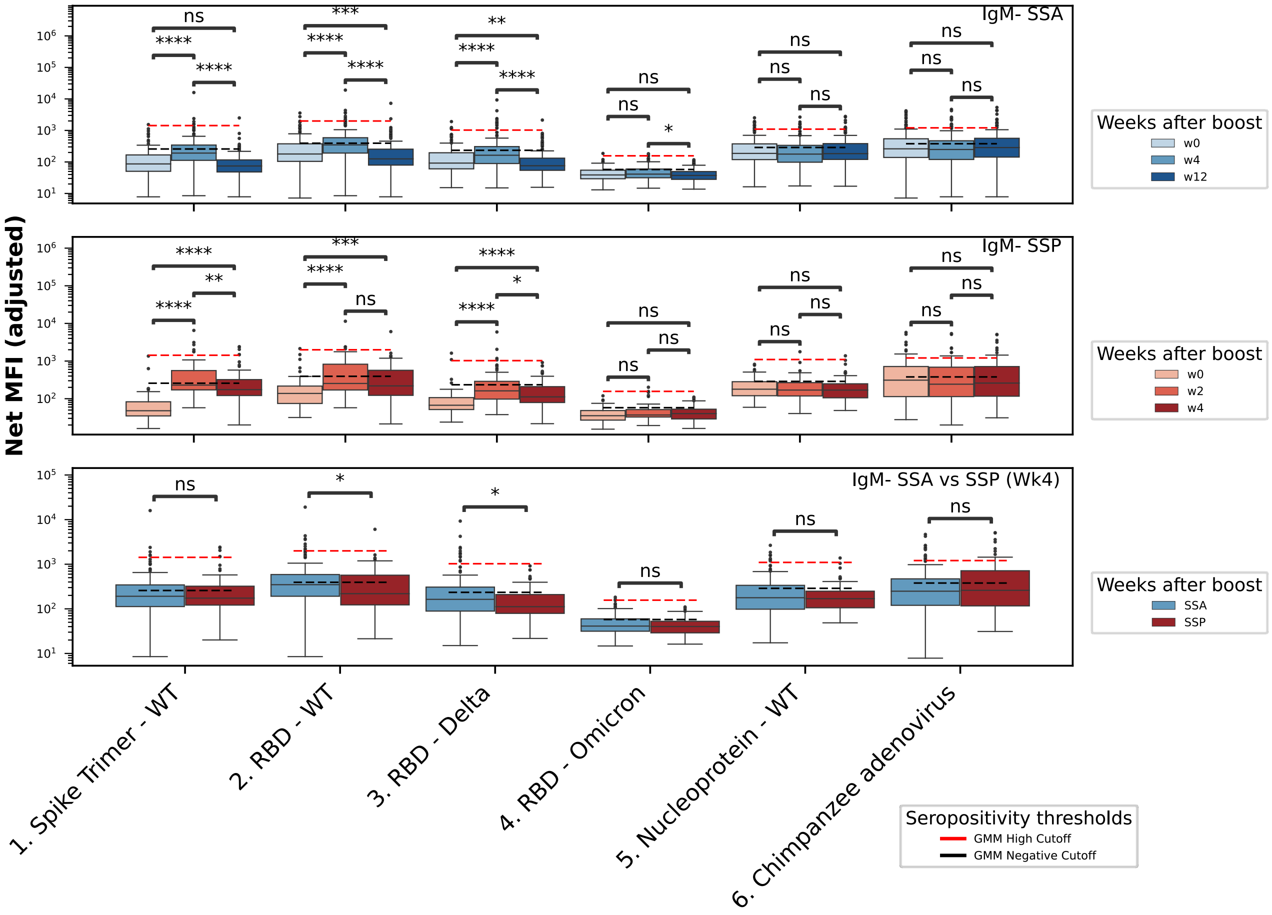

median fluorescence intensity (MFI)

(B) IgA (SSA top; SSP middle; SSA vs. SSP (at week 4) bottom)

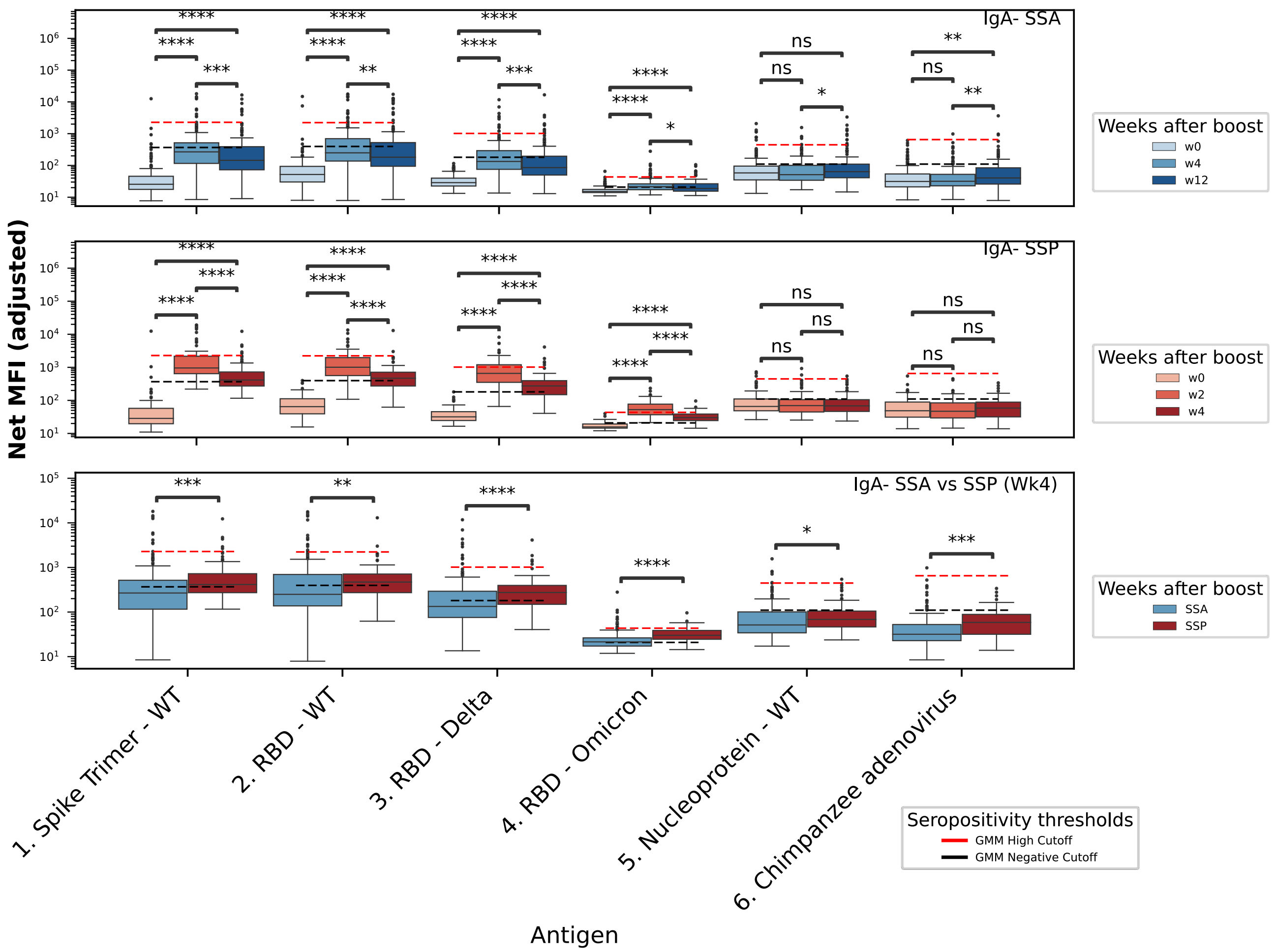

median fluorescence intensity (MFI)

(C) IgG avidity (SSA top; SSP middle; SSA vs. SSP (at week 4) bottom)

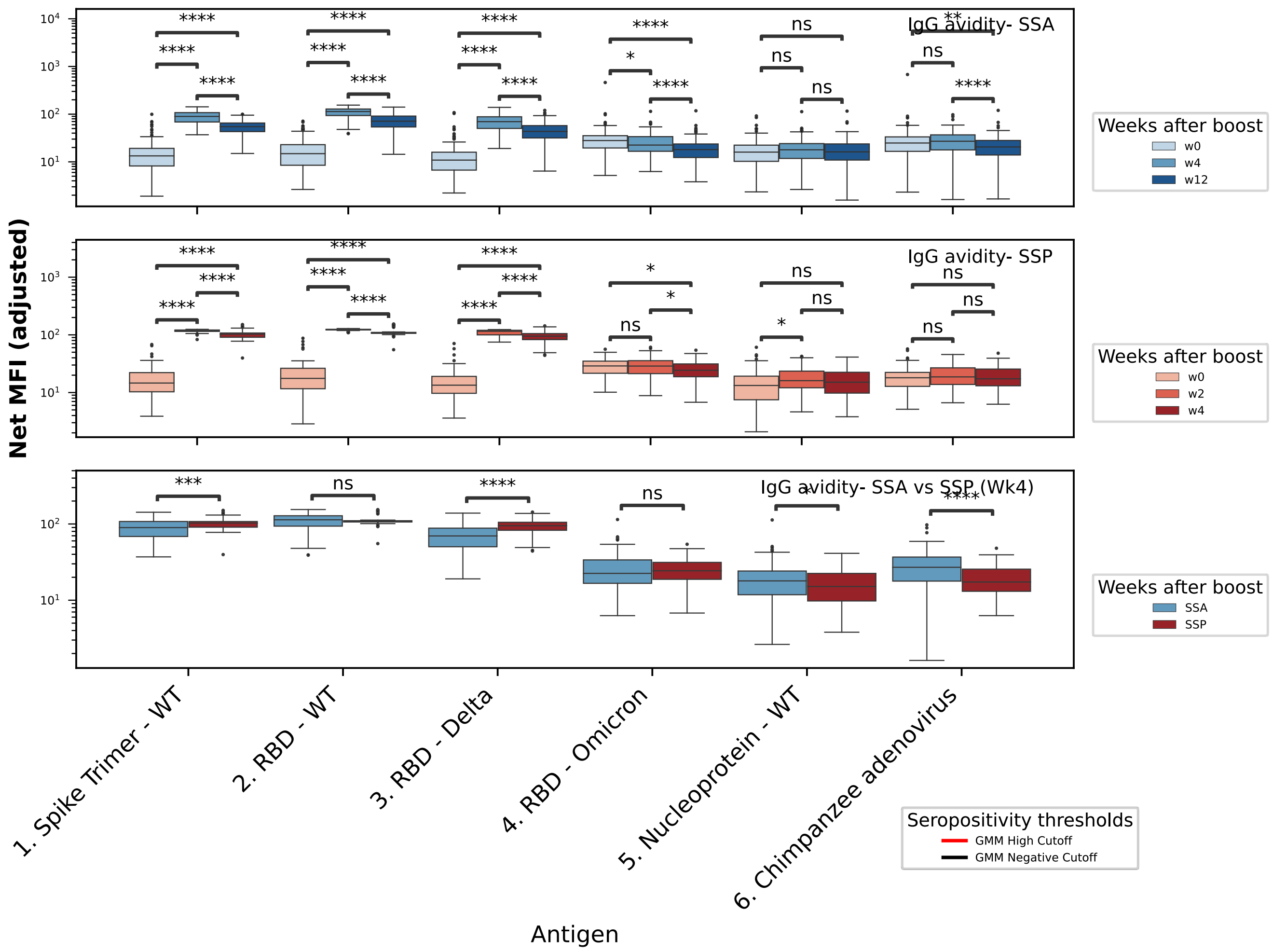

median fluorescence intensity (MFI)

Figure S4. The dynamics of anti-nucleocapsid IgG antibodies over 28 weeks since last Sinovac CoronaVac (S) vaccination.

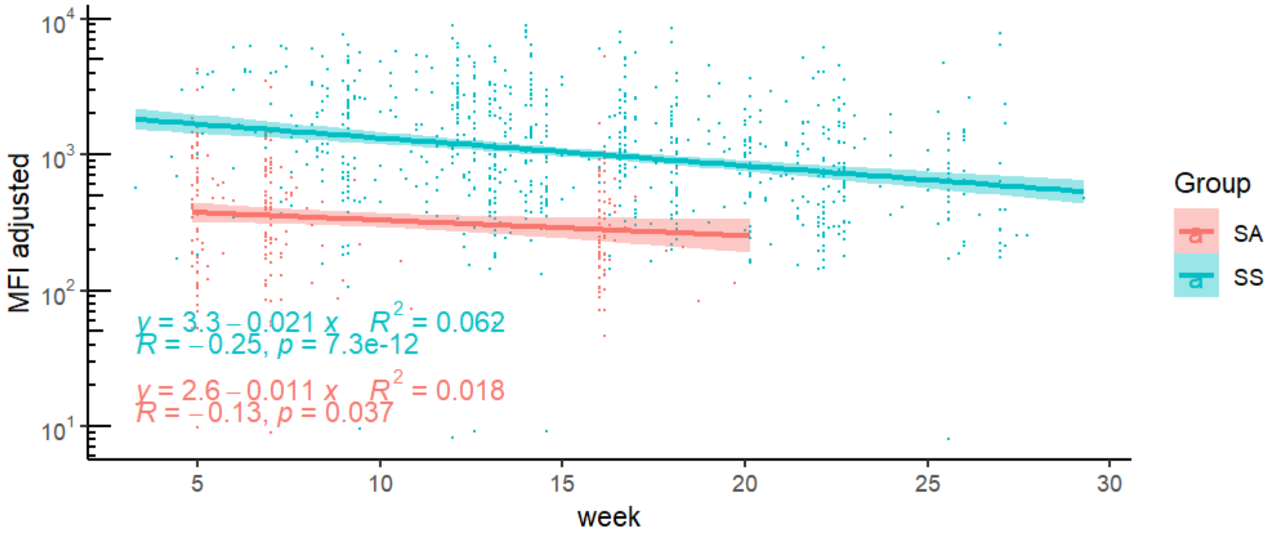

Figure S5. Examples of Gaussian mixture models (GMMs) applied to a frequency distribution of median fluorescence intensity (MFI) data across selected antigens and igG isotype. The Bayesian estimation of a Gaussian mixtures used a Dirichlet weighting distribution. Cut-offs were determined using the most probable label for each component.

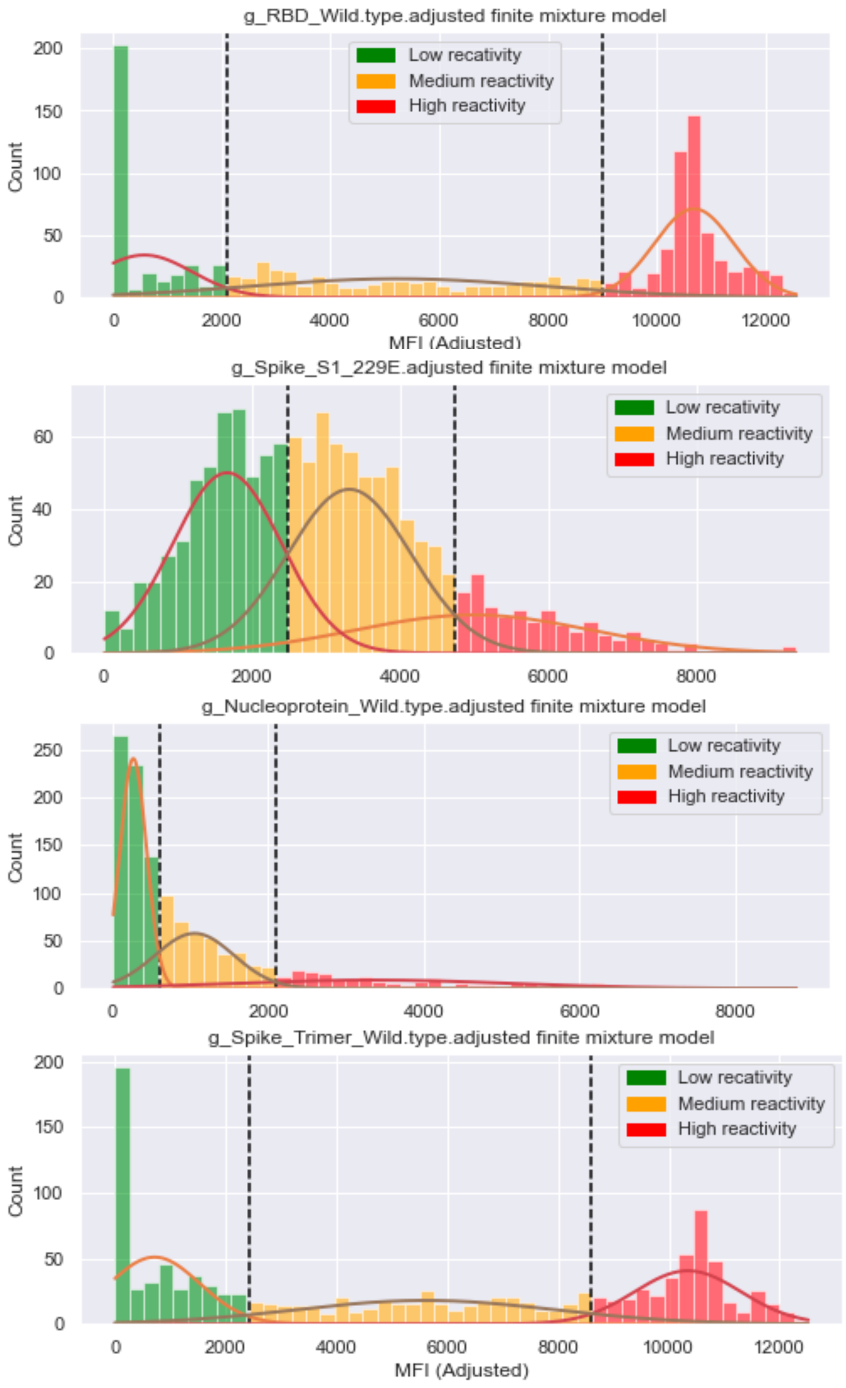

Supplementary tables

Table S1. Antigens used for the custom Luminex panel

Strain (Species)	Protein	Expression system	Coupling concentration/EC50 (µg/mL)
Wild-type	RBD	HEK293	5.1
Delta	RBD	HEK293	5.1
Omicron	RBD	HEK293	5.1
Wild-type	Spike Trimer	HEK293	2.7
Wild-type	Nucleoprotein	*E. coli*	3.7
Y25 adenovirus	Hexon	*E. coli*	10.0

RBD receptor-binding domain; HEK293 human embryonic kidney cells

Table S2. Median IgG, IgM, IgA and IgG avidity assay median fluorescence intensity (MFI) values (Figure 2)

(See **Supplementary tables** spreadsheet)

Table S3. Association between antibody responses across primary vaccine groups using a multivariate linear model*

Antibody	Antigen	Vaccine	Beta	95% CI	P-value
IgG	Spike_Trimer_WT	SA vs AA	0.098	(-0.018 - 0.214)	0.0994
IgG	Spike_Trimer_WT	SS vs AA	-0.454	(-0.578 - -0.331)	1.39E-11
IgG	RBD_WT	SA vs AA	0.114	(-0.011 - 0.239)	0.07482
IgG	RBD_WT	SS vs AA	-0.263	(-0.395 - -0.13)	0.00015
IgG	RBD_Delta	SA vs AA	0.123	(-0.007 - 0.253)	0.0648
IgG	RBD_Delta	SS vs AA	-0.410	(-0.548 - -0.272)	2.59E-08
IgG	RBD_Omicron	SA vs AA	-0.012	(-0.137 - 0.113)	0.854
IgG	RBD_Omicron	SS vs AA	-0.558	(-0.691 - -0.425)	3.15E-14
IgG	Nucleoprotein_WT	SA vs AA	0.270	(0.124 - 0.415)	0.000358
IgG	Nucleoprotein_WT	SS vs AA	0.918	(0.764 - 1.073)	<2e-16
IgG	Adenovirus_Hexon_ChAdOx	SA vs AA	-0.280	(-0.423 - -0.137)	0.000168
IgG	Adenovirus_Hexon_ChAdOx	SS vs AA	-0.390	(-0.542 - -0.238)	1.16E-06
IgG -Avidity	Spike_Trimer_WT	SA vs AA	-0.381	(-0.476 - -0.286)	3E-13
IgG -Avidity	Spike_Trimer_WT	SS vs AA	-0.673	(-0.774 - -0.573)	<2e-16
IgG -Avidity	RBD_WT	SA vs AA	-0.426	(-0.542 - -0.31)	1.69E-11
IgG -Avidity	RBD_WT	SS vs AA	-0.716	(-0.84 - -0.593)	<2e-16
IgG -Avidity	RBD_Delta	SA vs AA	-0.547	(-0.653 - -0.44)	<2e-16
IgG -Avidity	RBD_Delta	SS vs AA	-0.715	(-0.828 - -0.602)	<2e-16
IgG -Avidity	RBD_Omicron	SA vs AA	-0.277	(-0.358 - -0.195)	3.68E-10
IgG -Avidity	RBD_Omicron	SS vs AA	0.029	(-0.058 - 0.116)	0.509
IgG -Avidity	Nucleoprotein_WT	SA vs AA	0.044	(-0.06 - 0.148)	0.4042
IgG -Avidity	Nucleoprotein_WT	SS vs AA	-0.099	(-0.21 - 0.011)	0.0796
IgG -Avidity	Adenovirus_Hexon_ChAdOx	SA vs AA	0.111	(0.015 - 0.207)	0.0243
IgG -Avidity	Adenovirus_Hexon_ChAdOx	SS vs AA	0.135	(0.033 - 0.237)	0.0102
IgA	Spike_Trimer_WT	SA vs AA	0.413	(0.255 - 0.571)	7.97E-07
IgA	Spike_Trimer_WT	SS vs AA	-0.314	(-0.482 - -0.145)	0.000344
IgA	RBD_WT	SA vs AA	0.656	(0.496 - 0.817)	1.27E-13
IgA	RBD_WT	SS vs AA	-0.010	(-0.181 - 0.16)	0.904
IgA	RBD_Delta	SA vs AA	0.475	(0.355 - 0.594)	4.76E-13
IgA	RBD_Delta	SS vs AA	-0.043	(-0.17 - 0.084)	0.506
IgA	RBD_Omicron	SA vs AA	0.053	(0.009 - 0.096)	0.019
IgA	RBD_Omicron	SS vs AA	-0.026	(-0.072 - 0.02)	0.271
IgA	Nucleoprotein_WT	SA vs AA	-0.001	(-0.127 - 0.125)	0.98802
IgA	Nucleoprotein_WT	SS vs AA	0.100	(-0.034 - 0.234)	0.14622
IgA	Adenovirus_Hexon_ChAdOx	SA vs AA	0.152	(0.005 - 0.298)	0.0439
IgA	Adenovirus_Hexon_ChAdOx	SS vs AA	0.000	(-0.156 - 0.156)	0.998
IgM	Spike_Trimer_WT	SA vs AA	0.964	(0.821 - 1.106)	<2e-16

IgM	Spike_Trimer_WT	SS vs AA	0.310	(0.158 - 0.461)	9.04E-05
IgM	RBD_WT	SA vs AA	0.853	(0.686 - 1.02)	<2e-16
IgM	RBD_WT	SS vs AA	0.371	(0.193 - 0.548)	6.64E-05
IgM	RBD_Delta	SA vs AA	0.848	(0.690 - 1.006)	<2e-16
IgM	RBD_Delta	SS vs AA	0.340	(0.172 - 0.508)	0.000103
IgM	RBD_Omicron	SA vs AA	0.162	(0.068 - 0.257)	0.000917
IgM	RBD_Omicron	SS vs AA	0.032	(-0.069 - 0.133)	0.532704
IgM	Nucleoprotein_WT	SA vs AA	0.050	(-0.085 - 0.185)	0.46897
IgM	Nucleoprotein_WT	SS vs AA	0.163	(0.019 - 0.307)	0.02821
IgM	Adenovirus_Hexon_ChAdOx	SA vs AA	0.085	(-0.066 - 0.237)	0.27091
IgM	Adenovirus_Hexon_ChAdOx	SS vs AA	0.265	(0.104 - 0.426)	0.00151

* using log10 MFI values as the dependent variable, with adjustment for age, gender and interval time as covariates

Table S4. Median IgG, IgM, IgA and IgG avidity assay MFI values for the SSA and SSP vaccine regimen groups (Figure 2).

Antigen	Collection timepoint (week)	IgG Median (change)	IgM median	IgA Median	IgG avidity index Median
**CoronaVac-CoronaVac-ChAdOx1 nCoV-19 heterologous boost (SSA) MFI group**					
Spike Trimer - WT	0	1566.97	86.55	25.53	13.34
Spike Trimer - WT	4	10386.32 (>)	191.28 (>)	267.23 (>)	89.72 (>)
Spike Trimer - WT	12	8712.12 (<)	74.53 (<)	145.00 (<)	54.66 (<)
RBD – WT	0	2734.74	178.84	51.90	14.86
RBD – WT	4	10595.60 (>)	347.97 (>)	249.05 (>)	113.57 (>)
RBD – WT	12	10504.38 (<)	125.25 (<)	181.88 (<)	71.32 (<)
RBD - Delta	0	1126.29	92.12	28.77	10.88
RBD - Delta	4	9999.11 (>)	163.21 (>)	133.64 (>)	69.69 (>)
RBD - Delta	12	7825.20 (<)	75.83 (<)	85.61 (<)	43.47 (<)
RBD - Omicron	0	69.69	38.72	15.20	28.00
RBD - Omicron	4	1383.28 (>)	41.09 (>)	21.42 (>)	22.49 (<)
RBD - Omicron	12	613.15 (<)	37.26 (<)	18.87 (<)	18.04 (<)
Nucleoprotein - WT	0	1350.36	186.58	58.22	16.01
Nucleoprotein - WT	4	855.30 (<)	177.57 (<)	51.30 (<)	17.92 (>)
Nucleoprotein - WT	12	691.52 (<)	183.65 (>)	63.67 (>)	16.17 (<)
Chimp. adenovirus	0	65.92	260.93	31.32	24.84
Chimp. adenovirus	4	65.59 (<)	248.55 (<)	31.71 (>)	27.03 (>)
Chimp. adenovirus	12	90.97 (>)	286.46 (>)	40.36 (>)	20.54 (<)
**CoronaVac-CoronaVac-BNT162b2 heterologous boost (SSP) MFI group**					
Spike Trimer - WT	0	1269.79	47.88	28.49	14.61
Spike Trimer - WT	2	10591.40 (>)	229.95 (>)	956.94 (>)	118.36 (>)
Spike Trimer - WT	4	11606.62 (>)	173.88 (<)	413.47 (<)	102.76 (<)
RBD - WT	0	2113.59	137.90	64.61	17.61
RBD - WT	2	10608.98 (>)	253.84 (>)	1006.04 (>)	123.47 (>)
RBD - WT	4	11882.95 (>)	218.73 (<)	469.94 (<)	107.92 (<)
RBD - Delta	0	943.15	67.03	31.77	13.42
RBD - Delta	2	10475.88 (>)	161.48 (>)	656.10 (>)	115.39 (>)
RBD - Delta	4	11165.48 (>)	111.71 (<)	273.99 (<)	94.90 (<)
RBD - Omicron	0	71.10	35.33	15.71	28.92
RBD - Omicron	2	3777.18 (>)	36.91 (>)	52.54 (>)	28.80 (<)
RBD - Omicron	4	2899.72 (<)	40.15 (>)	30.05 (<)	24.32 (<)
Nucleoprotein - WT	0	1631.54	178.76	65.54	13.25
Nucleoprotein - WT	2	1250.88 (<)	169.67 (<)	69.44 (>)	16.06 (>)
Nucleoprotein - WT	4	1155.90 (<)	169.25 (<)	68.26 (<)	15.12 (<)
Chimp. adenovirus	0	97.25	311.01	48.61	18.05
Chimp. adenovirus	2	104.42 (>)	242.85 (<)	46.89 (<)	18.68 (>)
Chimp. adenovirus	4	100.78 (<)	260.22 (>)	58.47 (>)	17.32 (<)

Table S5. Geometric means of IgG, IgM, IgA and IgG avidity assay MFI values for the SSA and SSP vaccine regimen groups

Antigen	Collection timepoint (week)	IgG G. Mean (fold-change)	IgM G. mean (fold-change)	IgA G. mean (fold-change)	IgG avidity index G. mean (fold-change)
**CoronaVac-CoronaVac-ChAdOx1 nCoV-19 heterologous boost (SSA) MFI group**					
Spike Trimer - WT	0	1309.47	95.31	33.5	12.99
Spike Trimer - WT	4	9239.97 (7.06)	205.94 (2.16)	287.24 (8.57)	84.32 (6.49)
Spike Trimer - WT	12	7583.68 (0.82)	79.02 (0.38)	187.53 (0.65)	52.44 (0.62)
RBD – WT	0	2314.74	203.57	56.64	14.24
RBD – WT	4	9787.54 (4.23)	362.82 (1.78)	343.84 (6.07)	105.89 (7.44)
RBD – WT	12	9074.64 (0.93)	147.38 (0.41)	245.84 (0.71)	69 (0.65)
RBD - Delta	0	1046.04	114.93	31.69	10.78
RBD - Delta	4	8787.72 (8.4)	185.21 (1.61)	166.64 (5.26)	64.3 (5.96)
RBD - Delta	12	6995.47 (0.8)	90.09 (0.49)	111.51 (0.67)	40.34 (0.63)
RBD - Omicron	0	71.12	40.21	16.27	26.23
RBD - Omicron	4	1263.59 (17.77)	43.74 (1.09)	23.12 (1.42)	22.76 (0.87)
RBD - Omicron	12	613.03 (0.49)	38.37 (0.88)	20.94 (0.91)	17.62 (0.77)
Nucleo. - WT	0	1293.3	219.8	68.52	15.25
Nucleo. - WT	4	953.26 (0.74)	201.27 (0.92)	63.57 (0.93)	16.9 (1.11)
Nucleo. - WT	12	696.82 (0.73)	216.06 (1.07)	77.73 (1.22)	14.76 (0.87)
Chimp. Adeno.	0	67.5	276.99	38.03	23.06
Chimp. Adeno.	4	76.15 (1.13)	253.79 (0.92)	38.01 (1)	24.82 (1.08)
Chimp. Adeno.	12	94.22 (1.24)	298.71 (1.18)	49.12 (1.29)	18.89 (0.76)
**CoronaVac-CoronaVac-BNT162b2 heterologous boost (SSP) MFI group**					
Spike Tri. - WT	0	1288.2	55.75	40.09	15.09
Spike Tri. - WT	2	10679.71 (8.29)	319.45 (5.73)	1379.78 (34.42)	116.56 (7.72)
Spike Tri. - WT	4	11338.31 (1.06)	215.04 (0.67)	511.81 (0.37)	100.4 (0.86)
RBD - WT	0	2154.35	137.74	67.69	17.29
RBD - WT	2	10681.84 (4.96)	365.59 (2.65)	1105.1 (16.33)	122.18 (7.07)
RBD - WT	4	11568.16 (1.08)	261.7 (0.72)	486.33 (0.44)	111.1 (0.91)
RBD - Delta	0	1005.81	76.55	35.47	13.5
RBD - Delta	2	10471.01 (10.41)	186.56 (2.44)	648.73 (18.29)	109.24 (8.09)
RBD - Delta	4	10910.39 (1.04)	133.33 (0.71)	277.12 (0.43)	89.82 (0.82)
RBD - Omicron	0	80.3	36.94	17.01	27.77
RBD - Omicron	2	3722.23 (46.36)	41.6 (1.13)	54.67 (3.21)	27.56 (0.99)
RBD - Omicron	4	2829.86 (0.76)	40.21 (0.97)	31.14 (0.57)	23.94 (0.87)
Nucleo. - WT	0	1464.84	189.24	80.8	12.19
Nucleo. - WT	2	1223.6 (0.84)	175.91 (0.93)	80.8 (1)	16.12 (1.32)
Nucleo. - WT	4	1100.45 (0.9)	177.36 (1.01)	75.39 (0.93)	14.27 (0.89)
Chimp. Adeno.	0	102.94	313.45	52.92	17.29
Chimp. Adeno.	2	96.18 (0.93)	271.47 (0.87)	51.33 (0.97)	18.71 (1.08)
Chimp. Adeno	4	101.47 (1.05)	294.78 (1.09)	54.68 (1.07)	17.22 (0.92)

Table S6. Associations between Antibody responses and booster vaccine groups at week 4 using a multivariate linear model*

Antibody	Antigen	Vaccine	Beta	95% CI	P-value
IgG	Spike_Trimer_WT	SSP vs SSA	0.084	(-0.002 - 0.170)	0.0569
IgG	RBD_WT	SSP vs SSA	0.073	(-0.011 - 0.157)	0.0914
IgG	RBD_Delta	SSP vs SSA	0.086	(0.005 - 0.167)	0.0385
IgG	RBD_Omicron	SSP vs SSA	0.311	(0.226 - 0.396)	9.38E-12
IgG	Nucleoprotein_WT	SSP vs SSA	0.007	(-0.129 - 0.143)	0.9182
IgG	Adenovirus_Hexon_ChAdOx	SSP vs SSA	0.068	(-0.049 - 0.186)	0.2546
IgG -Avidity	Spike_Trimer_WT	SSP vs SSA	0.056	(0.018 - 0.094)	0.00414
IgG -Avidity	RBD_WT	SSP vs SSA	0.010	(-0.024 - 0.044)	0.559
IgG -Avidity	RBD_Delta	SSP vs SSA	0.133	(0.078 - 0.188)	4.01E-06
IgG -Avidity	RBD_Omicron	SSP vs SSA	0.015	(-0.053 - 0.083)	0.66
IgG -Avidity	Nucleoprotein_WT	SSP vs SSA	-0.100	(-0.183 - -0.018)	0.0177
IgG -Avidity	Adenovirus_Hexon_ChAdOx	SSP vs SSA	-0.144	(-0.222 - -0.066)	0.000365
IgA	Spike_Trimer_WT	SSP vs SSA	0.246	(0.082 - 0.411)	0.00363
IgA	RBD_WT	SSP vs SSA	0.197	(0.031 - 0.364)	0.0212
IgA	RBD_Delta	SSP vs SSA	0.250	(0.105 - 0.395)	0.000878
IgA	RBD_Omicron	SSP vs SSA	0.135	(0.078 - 0.193)	6.82E-06
IgA	Nucleoprotein_WT	SSP vs SSA	0.143	(0.032 - 0.254)	0.012
IgA	Adenovirus_Hexon_ChAdOx	SSP vs SSA	0.144	(0.037 - 0.251)	0.00893
IgM	Spike_Trimer_WT	SSP vs SSA	0.027	(-0.103 - 0.156)	0.689
IgM	RBD_WT	SSP vs SSA	-0.125	(-0.264 - 0.013)	0.0768
IgM	RBD_Delta	SSP vs SSA	-0.133	(-0.264 - -0.003)	0.0469
IgM	RBD_Omicron	SSP vs SSA	-0.080	(-0.142 - -0.018)	0.0125
IgM	Nucleoprotein_WT	SSP vs SSA	-0.109	(-0.222 - 0.005)	0.0617
IgM	Adenovirus_Hexon_ChAdOx	SSP vs SSA	-0.034	(-0.186 - 0.118)	0.66056

* using log10 MFI values as the dependent variable, with adjustment for age, gender, and time interval as covariates

Table S7. Component optimisation of Gaussian mixtures across number of features or dimensions of the model.

Dimen. *	Comp. **	Feature 1	Feature 2	Feature 3	Feature 4	F1-score
2	4	Trimer (IgG)	Nuc (IgG)			0.88
2	5	Trimer (IgG)	Nuc (IgG)			0.78
3	4	Trimer (IgG)	Nuc (IgG)	Trimer (IgG Avidity)		0.86
3	5	Trimer (IgG)	Nuc (IgG)	RBD Omicron (IgG)		0.82
4	4	Trimer (IgG)	Nuc (IgG)	RBD WT (IgM)	RBD Omicron (IgM)	0.86
4	5	Trimer (IgG)	Nuc (IgG)	RBD WT (IgG Avidity)	Trimer (IgG Avidity)	0.81

* Dimension is the number of features or isotype/antigen variables used; ** Components is the number of clusters/mixtures in the GMMs; Trimer = WT Spike Trimer, Nuc = WT Nucleoprotein IgG, WT = wildtype

Table S8. Buffers used for the Luminex assay

Buffer name	Component	Volume
**Buffer A (1x PBS-TBN)**	1x PBS	1000mL
	0.05% Tween	0.5mL
	0.5% BSA	5g
	0.02% Sodium azide	0.2g
**Buffer B (15.25 ug/mL E. coli lysate)**	Buffer A	1000mL
	0.1% casein	1g
	0.5% PVA	5g
	0.5% PVP	5g
	E. coli lysate (8.64 mg/mL)	1.77uL
**Activation Buffer**	NaH_2PO_4	1.2g
	MilliQ water	100mL
**Sulfo-NHS 50mg/mL**	Sulfo-NHS	0.05g
	MilliQ water	1000uL
**EDC 50mg/mL**	EDC	0.05g
	MilliQ water	1000uL
**Buffer B (15.25 ug/mL E. coli lysate)**	Buffer A	1000mL
	0.1% casein	1g
	0.5% PVA	5g
	0.5% PVP	5g
	E. coli lysate (8.64 mg/mL)	1.77uL
